# A Review of Embedded Machine Learning Based on Hardware, Application, and Sensing Scheme

**DOI:** 10.3390/s23042131

**Published:** 2023-02-14

**Authors:** Amin Biglari, Wei Tang

**Affiliations:** Klipsch School of Electrical and Computer Engineering, New Mexico State University, Las Cruces, NM 88001, USA

**Keywords:** computer vision, embedded systems, Google Coral, machine learning, Nvidia Jetson, RGB camera, Raspberry Pi, sensors

## Abstract

Machine learning is an expanding field with an ever-increasing role in everyday life, with its utility in the industrial, agricultural, and medical sectors being undeniable. Recently, this utility has come in the form of machine learning implementation on embedded system devices. While there have been steady advances in the performance, memory, and power consumption of embedded devices, most machine learning algorithms still have a very high power consumption and computational demand, making the implementation of embedded machine learning somewhat difficult. However, different devices can be implemented for different applications based on their overall processing power and performance. This paper presents an overview of several different implementations of machine learning on embedded systems divided by their specific device, application, specific machine learning algorithm, and sensors. We will mainly focus on NVIDIA Jetson and Raspberry Pi devices with a few different less utilized embedded computers, as well as which of these devices were more commonly used for specific applications in different fields. We will also briefly analyze the specific ML models most commonly implemented on the devices and the specific sensors that were used to gather input from the field. All of the papers included in this review were selected using Google Scholar and published papers in the IEEExplore database. The selection criterion for these papers was the usage of embedded computing systems in either a theoretical study or practical implementation of machine learning models. The papers needed to have provided either one or, preferably, all of the following results in their studies—the overall accuracy of the models on the system, the overall power consumption of the embedded machine learning system, and the inference time of their models on the embedded system. Embedded machine learning is experiencing an explosion in both scale and scope, both due to advances in system performance and machine learning models, as well as greater affordability and accessibility of both. Improvements are noted in quality, power usage, and effectiveness.

## 1. Introduction

Machine learning has become a ubiquitous feature in everyday life. From self-driving vehicles, facial recognition systems, and real-time interpretation of different languages, to security surveillance, smart home applications, and health monitoring, artificial intelligence has changed almost every society on earth [1,2,3,4]. Due to the extremely high computational requirements of machine learning models, until recently, the majority of these breakthroughs were implemented on high-power stationary computing systems. However, continuous advancements in embedded system design have made the implementation of machine learning models on embedded computing systems for a wide variety of mobile and low-power applications viable. One example of such an application would be [5], a 2020 paper by Ouyang et al., titled “Deep CNN-Based Real-Time Traffic Light Detector for Self-Driving Vehicles”, which proposes a method for recognizing traffic lights for autonomous vehicles. This ever-expanding research field of machine learning implementation in limited environments of embedded systems has been titled “Embedded Machine Learning” [6]. There are many considerations when choosing an embedded system for a specific machine learning application, such as power limitations, specific sensor outputs, model architecture, and monetary cost. In this review paper, we focus on the system models and assess which systems are better suited for which specific applications and sensing schemes.

As stated, machine learning algorithms are trained and used for many different applications, such as hand gesture recognition [7] and speech source identification [8]. They usually have a very high performance and memory requirement for both training and inference. Effective implementation would require the tuning and modification of the machine learning model architecture as well as the selection of the appropriate system depending on the priorities of the application. All machine learning applications aim to consume as little power and computation and be as fast and accurate as possible, however, improvement in one of these areas almost always comes at a relative cost to the other ones. Since embedded systems can vary drastically in power consumption, processing power, memory, storage, and pricing, it is prudent to select the appropriate system for each specific application. As an example, a system for pedestrian detection for autonomous vehicles [9] would prioritize performance speed and accuracy much more so than a system designed for recognizing marine life [10], even if it comes at a much higher monetary cost.

Training a machine learning model for any task requires a dataset, which can consist of megabytes to terabytes of images, video files, audio files, graphs, etc., and their corresponding annotation files. The specific files of a dataset used for training depend on the intended application of the machine learning model, an image classification model, for example, would use a dataset of image files and label annotations associated with each image. The sensing schemes used for collecting these files, both for the initial training and testing datasets, as well as for the inference of the trained machine learning algorithm on an embedded system, are varied. Another subject of analysis in this research was the correlation between the type of sensor scheme used in each system to the overall implementation of the system.

Most of the papers reviewed in this work utilized some form of computer vision, mainly in areas such as obstacle detection for autonomous vehicles (such as speed bumps) [11] or safety and security measures (such as violent assault identification) [12]. However, several also presented embedded machine learning methods for medical applications (such as patient heart monitoring) [13] or automating more aspects of city management (such as managing the direction and flow of vehicular traffic) [14].

**Figure 1:**
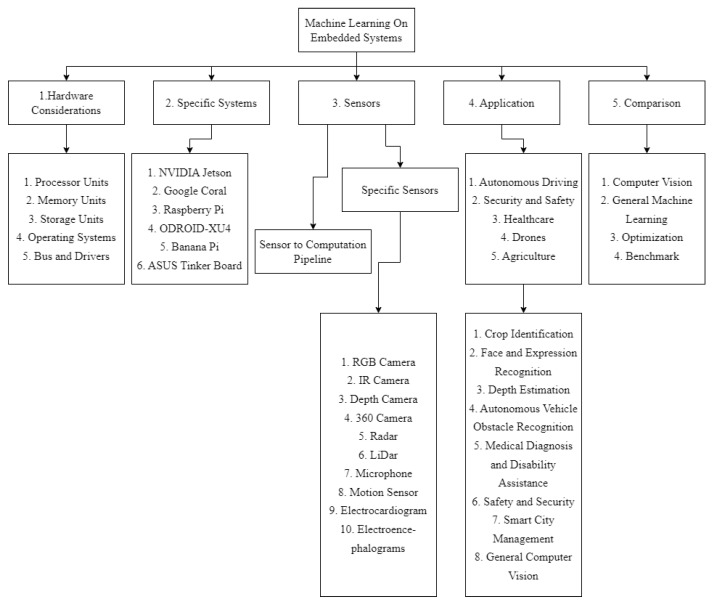
Paper Layout Showing the Distribution of Subjects Covered in the Review.

Essentially, in this review, we emphasized specific applications, embedded hardware platforms, and sensors, then compared them based on the nature of those networks and applications, while any other embedded machine learning review papers have a greater focus on the performance of specific lines of hardware [15], or the network architecture implemented on the hardware [16]. The paper is structured in the following format: 1. Abstract; 2. Introduction; 3. Hardware System Considerations; 4. Specific Hardware Systems Covered In The Review; 5. Sensing Systems; 6. Network Applications; 7. Comprehensive Comparisons; 8. Conclusions. This layout is also displayed in Figure 1. If the readers are interested in machine learning algorithms, models, and databases, please refer to other review and benchmark papers such as the ones used as sources in this work [15,16,17]. Works such as [18,19,20,21] and [15,17] provide a comprehensive performance analysis and benchmark of the embedded systems used in their specified applications, while works such as [22,23] conduct a more in-depth study on improvement methods for both system hardware and model architecture for their specific applications.

## 2. Objective and Method

To reiterate, the goal of this study is to summarize the current state-of-the-art research in the embedded machine learning area for different applications, so that the researchers could have an overview of the cutting-edge methods and results, as well predict the general trajectory of embedded machine learning advances. The method of research for this study was the compilation of the results gathered by the research papers referenced for this work. Excluding the related works in the Benchmark and Review section of the references, all of the papers presented in this review included a proposal or implementation of embedded machine learning for a specified application with the results of each study including one or all of the following findings: accuracy, inference speed, and power consumption.

## 3. Hardware

Embedded systems are computer hardware systems designed for performing dedicated functions in a combination with a larger system. They include and are used in many everyday items from mobile phones and household appliances. Embedded computer devices are a subset of embedded systems used for computational tasks for more dedicated or remote operations, such as running machine learning algorithms in real time on small unmanned aerial vehicles, connecting systems connected to the internet of things, and even security monitoring. While the variety of the embedded computer devices produced and used is quite wide, most academic research conducted on embedded machine learning is focused on using Raspberry Pi and NVIDIA Jetson devices. Some other devices used include the ASUS Tinker board series, Google’s Coral TPU dev series, ODROID-XU4 Boards, and the Banana Pi board series.

### 3.1. General Considerations

When choosing an embedded computing device for specific applications, many different parameters need to be kept in mind. The parameters include, but are not limited to, system processing speed, affected by the integrated CPU and GPU of a system, system memory affected by the RAM, system storage space, system bus and drivers, the overall power consumption of a system, and its cost of purchase. Generally, systems with higher performance and memory are capable of performing more complex machine learning tasks at a greater speed but have high power consumption rates and monetary prices. On the other hand, cheaper and less power-intensive systems have lower performances and memory, making them perform their dedicated task far slower.

### 3.2. Processor Units

Processing units are the integrated electrical circuits responsible for performing the fundamental algorithmic and arithmetic logic processes for running a computer device. There are different categories of processors, with the most common ones in embedded computer systems being CPUs and GPUs. Central Processing Units, or CPUs, are the processors present in most electrical devices and are responsible for the execution of programs and applications, they are usually composed of multiple cores and have their performance measured in gigahertz. Graphical Processing Units, or GPUs, are dedicated processors used for graphical rendering, allowing devices to allocate graphically intensive tasks, such as real-time object recognition, to them. All of the embedded computer devices presented in this review contain both a CPU and GPU unit, with the CPUs being various ARM Cortex multicore processors [24,25,26,27,28,29,30,31,32,33,34]. The GPUs for each system were more varied in both clock speed and power consumption. More detailed descriptions are given within each systems subsection.

### 3.3. Memory Units

System memory generally refers to a computing system’s Random Access Memory or RAM, which is responsible for storing application data for quick access. The larger a system’s RAM, the quicker the system can run simultaneous applications, making RAM proportional to the overall performance of a system. Embedded computing devices are packaged with their own memory component, with most embedded systems in this review having between 1 GB, 2 GB, and 4 GB of RAM [30,31], while the most recent NVIDIA kits have between 8 GB and 16 GB [24,28]. Memory Bandwidth is another important parameter of system memory, indicating the rate at which data can be accessed and edited, with the bandwidth of the system included in this review ranging from 128-bit to 256-bit.

### 3.4. Storage Units

Computer storage refers to the component of a computing device responsible for retaining longtime application and computation data. While access and alteration to storage data by the CPU are much slower than its access to RAM data, it consumes far less power and processing capability. Storage systems come in many varieties such as flash drives, hard drives, solid state drives, SD cards, and embedded MultiMediaCard memory or eMMC. Hard drives have been the most common form of storage up until recently, with their advantage over other alternatives being their overall size and their downside being their relatively slow data access speed. Solid state drives or SSDs have provided far faster data access at the cost of storage size, however, in recent years, SSDs have made leaps in storage capacity and are now comparable in overall storage size to hard drives. Flash drives are quick and easy to connect or disconnect from different computing devices while having very small storage space, they are very similar to SSDs in terms of performance. Secure digital cards or SD cards are also similar to flash storage but have both much smaller sizes and storage capacities. eMMCs are architecturally similar to flash storage and are generally used in small laptops and embedded computing systems. Most development kit embedded computing systems contain eMMCs, this being very much the case in NVIDIA Jetson, Coral Edge, and ASUS Tinker board devices, and others, such as ODROID-XU4 boards, do not have their own integrated storage devices but instead have flash storage interface. Raspberry Pi boards have interfaces for both SD cards and Flash drives.

### 3.5. Operating Systems

Operating systems are responsible for managing and running all of the applications on a computing device, allowing applications to make requests for services through a defined application program interface (API). This makes the creation and usage of various applications much simpler, as all low-level functions, such as allocating disk space for an app, can be delegated to the OS. Operating systems rely on a library of device drivers to their services to specific hardware environments, so while every application makes a common call to a storage device, it is the OS that receives that call and uses the corresponding driver to translate the call into commands needed for the underlying hardware. Hardware capabilities are divided into three sections: providing UI through a CLI or GUI, launching and managing application execution, and identifying and exposing system hardware resources to the applications. Most personal computing devices utilize general-purpose operating systems, such as Windows, Mac OS, and Linux, and while there are specific embedded operating systems, mainly used in ATMs, Airplanes, and ioT devices, most embedded computing systems either utilize operating systems based on or very similar to general-purpose computer operating systems. For example, Nvidia Jetson boards have Linux for Tegra included in their development software kits [35].

### 3.6. Bus and Drivers

Computer buses are communication systems responsible for transferring data between the various components of a computing system. While most home computer systems have 32-bit to 64-bit buses, embedded devices have far smaller bit rates between 4-bit and 8-bit. Drivers refer to the systems responsible for communicating the software of a computer device to its hardware component. They generally run at a high privilege level in the OS run time environment, and in many cases are directly linked to the OS kernel, which is a portion of an OS such as Windows, Linux, or Mac OS, which remains memory-resident and handles execution for all other code. Drivers are what defines the messages from the OS to a specific device that facilitate the devices’ fulfillment of the OS’s request. The device drivers used in each embedded computing system are related to the operating systems of each device. For example, Raspberry Pi devices mainly use Raspberry Pi’s own operating system which is based on Debian, while Nvidia Jetson boards mainly rely on JetPack, Nvidia’s proprietary Software Development Kit (SDK) for their Jetson board series, which includes the Linux for Tegra (L4T) operating system. This means the driver kernels for both of these embedded system product lines are similar to that of a Linux computer [36].

Firmware refers to software formats that are directly embedded in specific devices, giving users low-level control over them. Essentially, firmware is responsible for giving simple devices their operation and system communication instructions. They are unique to other software in that they do not rely on APIs, OSs, or device drivers for operation. They are the first part of device programming to start sending instructions when the device is powered on, and in some more simple devices such as keyboards, they never pause their operations. They are mostly installed on a ROM for software protection and proximity to the physical component of their specific device. They can only work with a basic or low-level binary language known as machine language [37]. All of this applies to the components within an embedded system, meaning each device within the system has its own unique firmware with varying levels of complexity based on the function of the device.

## 4. Specific Systems

### 4.1. Nividia Jetson

Jetson is the name of a series of machine learning embedded systems by NVIDIA used for autonomous devices and various embedded applications. While Jetson Developer kits vary in capability and performance, they are generally very reliable for implementing machine learning tasks—this is especially true for more graphically intensive applications. The downside to this is that NVIDIA Jetson boards also tend to be more costly than market alternatives. Most of the sources shown in this review either only made use of Jetson boards or used their combination with other devices. These specific developer kits were the NVIDIA Jetson Nano, NVIDIA Jetson TX1, NVIDIA Jetson TX2, NVIDIA Jetson AGX Xavier, and NVIDIA Jetson Xavier NX.

NVIDIA Jetson Nano is one of the smaller Jetson kits specialized for machine learning tasks like image classification, object detection, segmentation, and speech processing. It has a 128-core Maxwell GPU, a Quad-core ARM Cortex A57 1.4Remote Sensing of EnvironmentHz CPU, 4 GB 64-bit LPDDR4 25.6 GB/s Memory, 2x MIPI CSI-2 DPHY lanes camera, Ethernet, HDMI, and USB connection ports. Unlike most other NVIDIA kits, Nano does not have an integrated storage unit and has to rely on SD cards for that purpose. It has a power consumption of 5–10 Watts and with a price range of USD 300–USD 500, it is the more affordable option out of all of the NVIDIA development kits [24].

The Jetson TX1 and TX2 series are a discontinued line of embedded system development kits with flexible capabilities that include great performance for machine learning tasks. As the discontinuation of this line of kits is especially recent for the TX2 series, research publications that utilize the TX2 board are not uncommon, with the TX1 being much rarer. The TX1 has a 256-core Maxwell GPU, a Quad-core ARM® Cortex®-A57 CPU, a 4 GB LPDDR4 memory, a 16 GB eMMC 5.1 Flash Storage, a 5 MP Fixed Focus MIPI CSI Camera, Ethernet, HDMI, and USB type A and Micro AB connection ports. The TX2 has NVIDIA Pascal™ Architecture GPU, 2 64-bit CPUs, Quad-Core Cortex®-A57 Complexes, an 8 GB L128 bit DDR4 memory, a 32 GB eMMC 5.1 Flash Storage, a 16 GB eMMC 5.1 Flash Storage, a 5 MP Fixed Focus MIPI CSI Camera, and Ethernet, HDMI, and USB type A and Micro AB connection ports. The power consumption of the TX1 is around 15 Watts and that of the TX2 is about 25 Watts [25,26].

The Jetson AGX Xavier is one of the most powerful developer kits produced by NVIDIA. It is mainly used for creating and deploying end-to-end AI robotics applications for manufacturing, delivery, retail, and agriculture, but it could also be applied for less intensive machine learning applications. It has a 512-core Volta GPU with Tensor Cores, an 8-core ARM v8.2 64-bit CPU, a 32 GB 256-Bit LPDDR4x memory, a 32 GB eMMC 5.1 Flash storage, as well as two USB C ports, and an HDMI and camera connector. It has a price of about USD 4000 and has a power consumption of 30 Watts, making it much more costly in both price and electricity than the other Jetson kits [27].

The Jetson Xavier NX kits is another series of NVIDIA developer kits designed as the successor to the TX series. It is power-efficient and compact, making it suitable for machine learning application development. It has an NVIDIA Volta architecture GPU with 384 NVIDIA CUDA® cores and 48 Tensor cores, a six-core NVIDIA Carmel ARM®v8.2 64-bit CPU, an 8 GB 128-bit LPDDR4x memory, two MIPI CSI-2 DPHY lanes cameras, and Ethernet, HDMI, and USB type A and Micro AB connection ports. It has an integrated storage component of its own, instead of relying on a micro SD storage interface. It has a power consumption of 10 Watts and a price range of around USD 2000. Its well-rounded quality makes it a very good, if somewhat expensive, the choice for machine learning implementation on embedded systems [28].

### 4.2. Google Coral

Google Coral Dev Board is a single-board computer by Coral that can be used to perform fast machine learning (ML) inferencing in a small form factor; it is mainly used for prototyping custom embedded systems, but it can also be used for embedded machine learning on its own. It has an Edge TPU coprocessor that is capable of performing 4 trillion operations per second, as well as being compatible with TensorFlow Lite. It has a quad Cortex-A53 CPU, integrated GC7000 Lite Graphics, 1 GB/2 GB/4 GB LPDDR4 memory, 8 GB eMMC storage as well as a MicroSD slot, Type C, A, and microB USB, Gigabit Ethernet, and HMDI 2.0 ports. The overall board has a low power cost of 6–10 Watts and at USD 130, the price for the board is relatively low [29].

### 4.3. Raspberry Pi

Raspberry Pi is a series of extremely popular embedded computers developed by the Raspberry Pi Foundation in the United Kingdom. The uses for these systems are extremely wide, including machine learning. Like the Jetson series, Raspberry Pi products are very commonly used in embedded machine-learning implementation projects. For this review, the three systems of Raspberry Pi that were commonly utilized were the Raspberry Pi 3 Model B, the Raspberry Pi 3 Model B+, and the Raspberry Pi 4 Model B.

The Raspberry Pi 3 Model B is the first iteration of the third-generation Raspberry Pi computers. It has a Quad Core 1.2 GHz Broadcom BCM2837 64bit CPU, a 400 MHz VideoCore IV video processor, a 1 GB LPDDR2 memory, a microSD port for storage, a 100 Base Ethernet, 4 USB 2.0, and full-size HDMI ports. It has an extremely low power consumption of 1.5 Watts and a monetary cost of about USD 40 [30].

The Raspberry Pi 3 Model B+ is the final iteration of the third-generation Raspberry Pi Computers. It has a Quad Core 1.4 GHz Broadcom BCM2837B0, Cortex-A53 (ARMv8) 64-bit SoC CPU, a 400 MHz VideoCore IV video processor, a 1 GB LPDDR2 memory, a microSD port for storage, a 1000 Base Ethernet, 4 USB 2.0, and full-size HDMI ports. Its main advantage to model 3b is its processor’s higher clock speed and its PoE (power over Ethernet) support. At 2 Watts, its power consumption is still low but higher than that of the model 3b series. It also has a very close monetary cost ranging around USD 40.

The Raspberry Pi 4 Model B is the first iteration of the fourth-generation Raspberry Pi Computer. It has a Quad Core 1.5 GHz Broadcom BCM2837B0, Cortex-A72 (ARMv8) 64-bit SoC CPU, a 400 MHz VideoCore IV video processor, a choice between 1 GB, 2 GB, 4 GB, and 8 GB LPDDR2 memory, a microSD port for storage, a Gigabit Ethernet, 4 USB 2.0, and full size HDMI ports. Its main advantage to model 3b is its processor’s higher clock speed and its PoE (power over Ethernet) support. Its newer processor and option for memory make it a superior choice compared to the previous iteration of Raspberry pi. It has a relatively low power consumption of 4 Watts and a monetary cost of about USD 40–USD 80 depending on the memory size [31].

### 4.4. ODROID XU4

The ODROID XU4 is an energy-efficient single-board embedded computing system by Hardkernel Co. located in Rm704 Anyang K Center 1591-9 Gwanyang-dong Dongan-gu, Anyang-si, Gyeonggi-do, South Korea. It is compatible with open-source software and can use different versions of Linux, such as Ubuntu, as its operating system. It has Exynos5422 Cortex™-A15 2 Ghz and Cortex™-A7 Octa core CPUs, a Mali-T628 MP6 GPU, a 2 GB LPDDR3 memory, 2 GB eMMC5.0 LPDDR3 Flash Storage as well as a microSD slot, 2 USB 3.0 and 1 USB 2.0, Gigabit Ethernet, and HMDI 1.4 ports. It has an operating power of 5 Watts and its cost is generally around USD 100 [32].

### 4.5. Banana Pi

Banana Pi is an open-source hardware platform by Shenzhen SINOVOIP Co. located in 7/F, Comprehensive Building of Zhongxing Industry City, Chuangye Road, Nanshan District, Shenzhen, China. Like other embedded systems, it has a wide range of applications, amongst them, embedded machine learning implementation. It has an H3 Quad-core Cortex-A7 H.265/HEVC 4K, a Mali400MP2 GPU, 1 GB DDR3 Memory, an 8 GB eMMC Onboard Storage, two USB 2.0 ports, an HDMI port, and an Ethernet interface. Its overall power consumption is about 5 Watts and it has a price range of USD 50–USD 75 [33].

### 4.6. ASUS Tinker Board

The ASUS Tinker Board S is a powerful SBC board with a wide range of functions such as computer vision, gesture recognition, image stabilization, and processing, as well as computational photography. It has a Rockchip Quad-Core RK3288 CPU, an ARM® Mali™-T764 GPU, a 2 GB Dual-Channel DDR3 Memory and 16 GB eMMC Onboard Storage 4 USB 2.0, and an Ethernet port, and RTL GB LAN connectivity. It has a maximum power consumption of 5 Watts and is a relatively low-price system for all of its capabilities ranging in price from USD 100–USD 150 [34].

The ASUS Tinker Edge R is specifically developed for AI applications, containing an integrated Machine Learning (ML) accelerator that speeds up processing efficiency, lowers power demands, and makes it easier to build connected devices and intelligent applications. It has an Arm® big.LITTLE™ A72+A53 Hexa-core CPU, an ARM® Mali™-T860 MP4 GPU, a 4 GB Dual-CH LPDDR4 memory on its system, and a 2 GB LPDDR3 on the Rockchip NPU, a 16 GB eMMC Flash Storage as well as a microSD slot, 3 USB 3.2 type A and 1 USB 3.2 Type C, Gigabit Ethernet, and HMDI ports. It can maintain a maximum power supply of 65 Watts and is a relatively lo- price system for all of its capabilities ranging in price from USD 200–USD 270 [38].

All of the inforamtion related to hardware specification has been summarised in Table 1.

## 5. Sensors

Electrical sensors are components responsible for gathering input from a given physical environment. The specific input that a sensor responds to varies from sensor to sensor could be temperature, ultrasound waves, light waves, pressure [39,40], or motion. Sensors do this by acting as switches in a circuit, controlling the flow of electric charges through their overall systems. Sensors can be split into two separate overarching categories, active sensors, and passive sensors. Active sensors emit their own radiation such as ultrasound waves and laser, from an internal power source, which is then reflected from the objects in the environment, the sensor then detects these reflections as inputs. radars are an example of active sensors. Passive sensors simply detect the radiation or signature emitted from their targets, such as body heat [41].

The most important characteristics of sensor performance are transfer function, sensitivity, span, uncertainty, hysteresis, noise, resolution, and bandwidth. The transfer function shows the functional relationship between the physical input signal and the electrical output signal. The sensitivity is defined in terms of the relationship between the input physical signal and the output electrical signal. The span is the range of input physical signals that may be converted to electrical signals by the sensor. Uncertainty is generally defined as the largest expected error between actual and ideal output signals. Hysteresis is the width of the expected error in terms of the measured quantity for sensors that do not return to the same output value when the input stimulus is cycled up or down. Output noise is generated by all sensors in addition to the output signal, and since there is an inverse relationship between the bandwidth and measurement time, it can be said that the noise decreases with the square root of the measurement time. The resolution is defined as the minimum detectable signal fluctuation. The bandwidth is the frequency range between the upper and lower cutoff frequencies, which respectively correspond to the reciprocal of the response and decay times [42].

Once sensors acquire input and convert it into electrical current, they can communicate their data to the rest of an overarching system through a variety of means, the main methods being to transfer data over a wired interface, or transfer data wirelessly [43,44]. Since the embedded systems studied in this research all made use of wired communication for their sensing systems, we focus only on analog communication. Standard wired interfaces between sensors and computing devices use serial ports, which transfer data between the data terminal equipment (DTE) and data circuit-terminating equipment (DCE). For successful data communication, the DTE and DCE must agree on a communication standard, the transmission speed, the number of bits per character, and whether stop and parity framing bits are used. Most modern-day computing devices and embedded systems use USB standards for their communication, connection, and power peripherals, which includes any additional sensor systems. USBs have had many port-type iterations since their inception; USB 1.x (up to 12 Mbps speed), USB 2.0 (up to 480 Mbps speed), USB 3.0 (up to 5 Gbps speed), and USB4 (super speed, up to 40 Gbps), most devices have ports for the USB 2.0 and USB 3.0 port types, with the USB4 being mostly suited for mobile smartphone devices. One of the main advantages of USB devices, including sensor systems, is that they can have multiple functionalities through a single connection port, for example, a USB camera can record both video and audio. These devices are referred to as composite devices and each of their functionalities is assigned to a specific address. USB devices can draw 5V and a maximum of 500mA from a USB host, allowing both data interface for sensor systems as well as powering the sensor component [45].

### 5.1. Sensor-to-Computation Pipeline

Once sensor systems receive input, they convert the input into digital data and transfer it to a display or a larger system. The format of the gathered data depends on the specific input a sensor collects, cameras would collect videos or images and microphones would collect audio. The environmental data collected by sensors are then stored within internal or external storage components connected to the overall system. These data are then used for whatever purpose the overall system that employed the sensor has been designed for.

As the focus of these research projects is over-viewing the capability of different embedded systems for running machine learning models, all of the sensor data are transferred to a previously trained machine learning algorithm or used to train a new algorithm based on existing architecture. In cases of trained model deployment, depending on the exact application of the model as well as its architecture, the stored data collected by the sensor systems is transferred to the model to perform predictions. For example, image identification and object recognition models will compare images files to the dataset images they have been trained with to either identify the specific objects of interest or the entire image, while forest biomass estimation models would compare the results gathered from lidar sensors to their trained dataset to estimate the concentration of vegetation in certain areas of forests [46].

### 5.2. Specific Sensors

Much like the different embedded computing systems that were used for machine learning implementation, many different sensors were used in each of our review sources depending on the application of the research. Not all sources made active use of a sensor within their work, and mainly explored the theoretical implementation of their machine-learning models using sensor systems. Amongst those that did implement their systems in some capacity, many implemented some form of object detection, image recognition, image segmentation, and other forms of computer vision, making extensive use of different integrated and separate image and video cameras. These cameras included infrared, RGB, Depth, Thermal, and 360-degree cameras. Other sensors used included microphones, electrocardiograms, radar, motion sensors, LIDAR, and multi-sensors.

#### 5.2.1. RGB Cameras

RGB color cameras or visible imaging sensors are sensor systems that collect and store visible light waves as electrical signals that are then reorganized as rendered colored images. The images and videos they capture replicate human vision, capturing lightwave with (400–700) nm wavelength through light-sensitive electrical diodes, then saving them as pixels. Modern-day cameras can capture high-definition images [47]. The main use of these sensors is for object detection and image classification algorithms. Among the sources in this review, the main application in which an RGB camera was implemented included autonomous vehicles for pedestrian and sign detection, security cameras for intruder detection, facial recognition, and employee safety monitoring, and drones for search and rescue, domestic animal monitoring [48,49], agricultural crops, and wildlife observation [50].

#### 5.2.2. Infrared Cameras

Infrared cameras or thermal imaging sensors are sensor systems that collect and store the heat signature that is emitted from objects as electronic images that show the apparent surface temperature of the captured object. They contain sensor arrays, consisting of thousands of detector pixels arranged in a grid on which infrared energy is focused. The pixels then generate an electrical signal that is used to create a color map image corresponding to the heat signature detected on an object ranging from violet to red, yellow, and finally white, with deep violet corresponding to the lowest detected heat signature and bright white corresponding to the highest detected heat signature [51]. In a similar sense to RGB cameras, the main use of these sensors is for object detection and image classification algorithms, albeit for more specialized tasks. Applications proposed by the sources in this review included autonomous vehicles for pedestrian detection, hand gesture, sign language, and facial expression recognition, thermal monitoring of electrical equipment, and profile recognition in smart cities.

#### 5.2.3. Depth Cameras

Depth or range cameras are specific forms of sensor systems used to measure the exact three-dimensional depth of a given environment. They work by illuminating the scene with infrared light and measuring the time-of-flight. There are two operation principles for these sensors, pulsed light, and continuous wave amplitude modulation. In a sense, depth camera operation is very similar to Lidar, with it relying on infrared radiation reflection instead of laser [52]. The main application depth cameras used in among the sources of this paper were for quad-copter drone formation control, ripe coffee beans identification, and personal fall detection.

#### 5.2.4. 360 Degree Cameras

360-degree cameras are sensor systems used to record images or video from all directions in 3D space using two over-180-degree cameras facing the front and rear of the device, the borders of the two images or videos are then stitched together to create a seamless single 360 image or video file. Users and automated applications can then select a specific section of the captured 360-image or footage for the intended use. Other than the over 180-field of view for each camera lens, 360 cameras work in an identical fashion to RGB cameras capturing visible spectrum light and storing it as digital data in pixel format [53,54]. While 360 cameras have various applications, from recreational ones such as vlogging and nature photography to navigational ones such as Google Maps, the sources used in this paper mainly relied on them for biometric recognition and marine life research.

#### 5.2.5. Radar

RADAR, short for Radio Detecting And Ranging, is a radio transmission-based sensor system designed for detecting objects. They operate using short-pulse electromagnetic waves, these pulses are then reflected from objects in the path of the RADAR sensor and are then reflected back at it. Essentially, “When these pulses intercept precipitation, part of the energy is scattered back to the RADAR” [55]. RADAR systems can rely on 14 different frequency bands depending on the application. RADAR systems have a wide variety of applications, from meteorology to military surveillance and astronomical studies. Among the sources used for this review, RADAR systems were scarcely used, and within these cases, the main usage was for electric hybrid car deep learning-based car following systems as well as multi-target classification for security monitoring.

#### 5.2.6. LiDar

Lidar (light detection and ranging) sensors are sensor systems that emit millions of laser waveforms and then collect their reflection to precisely measure the shape and distance of physical objects in a 3D environment. Essentially, they are laser-based radar systems. This process is repeated millions of times per second to create a precise real-time three-dimensional map of an area called a point cloud, which can then be used for navigation systems [56]. While the technology itself is decades old, with improvements in Lidar performance in terms of range detection, accuracy, power consumption, as well as physical features such as dimension and weight, its popularity has been rising in recent years, especially in the fields of robotics, navigation, remote sensing, and advanced driving assistance [57]. Lidars’ main usage among our sources was for locating people in danger in search and rescue operations, such as one following an earthquake, and optimizing trajectory tracking for small multi-rotor aerial drones.

#### 5.2.7. Microphones

Microphones are sound sensors that act as transducers, converting sound waves into electrical current audio signals carrying the sound data. When sound waves interact with the microphone diaphragm, the vibrations created are converted into a coinciding audio signal via electromagnetic or electrostatic principles that will be outputted [58]. This audio signal can then be stored as digital data and replayed or used in other applications such as training sound recognition machine learning models. The sources presented in this review mainly used microphones for real-time speech source localization.

#### 5.2.8. Body Motion Sensors

Body motion sensors, also known as motion capture sensors, are a series of sensor systems that are used to keep track of a person or a physical movement or physical posture. They generally work by making use of other sensing systems, including photosensors, angle sensors, IR sensors, optical sensors, accelerometers, inertial sensors [59], and magnetic bearing sensors [60]. Mocap sensors have been widely known for their use in the entertainment industry, but with recent advances, they have become more affordable and accurate for common consumer use. The application for which motion capture was used among the sources in this review is complex posture detection.

#### 5.2.9. Electrocardiograms

Electrocardiograms are heart monitoring sensors used for quick analysis of a patient’s heart [61,62,63]. Heart contractions generate natural electrical impulses that are measurable by nonintrusive devices, such as lead wires placed on a patient’s skin. The measured pulses are then converted into an electric signal that can be used to measure irregularities in the patient’s heart rate [64]. Naturally, electrocardiograms are mainly used in medical facilities or by caregivers and nurses to monitor heart health [65,66], however, the sources used for this review have also utilized them for identifying epileptic seizures.

#### 5.2.10. Electroencephalograms

Electroencephalograms are brain monitoring sensors used for analyzing a patient’s brain activity. The brain’s processes are the result of electrical current traveling through its neurons at varying levels depending on the current state of a patient, what they are doing, or how they are feeling. Electroencephalograms record these currents across the various brain regions using painless electrodes placed around a patient’s scalp. These fluctuations recordings are then saved as either a paper or digital graph [67]. Much like electrocardiograms, electroencephalograms are mainly used in medical facilities or by caregivers and nurses to monitor heart health, however, sources used for this review have also utilized them for anesthesia patient monitoring.

## 6. Applications

Embedded machine learning applications are all either of a remote nature or require more mobile systems to be implemented. The applications which are covered in this review are divided into the following categories: autonomous driving, security, personal health and safety, unmanned aerial vehicle navigation, and agriculture.

### 6.1. Autonomous Driving

Autonomous driving refers to the ever-expanding field of assisted and self-driving vehicles. It involves the implementation of a machine learning algorithm designed to detect obstacles, street signs, pedestrians, and other vehicles. Almost all self-driving vehicle AI models are computer vision models such as object and depth detection and distance measurement, with some exceptions that rely on Lidar or Radar for obstacle detection. Due to the nature of the application, the highest priority for models developed on embedded systems for self-driving vehicles is performance speed. Driving requires extremely short reaction time and that makes the speed at which a model can identify objects and allow the other car systems to make driving decisions very important.

### 6.2. Security and Safety

Security applications of machine learning can be related to many different sections such as intruder detection or personnel safety in hazardous worksites [68]. Once again, most of these models are trained for computer vision purposes in order to identify different individuals and ensure authorized access to secure locations and information. They do this through facial recognition and biometric identification using embedded system-operated camera systems, to name a few avenues. Ensuring personnel safety in hazardous work environments also involves constant monitoring by camera systems, to see if any of the employers are showing visible signs of illness or injury. Accuracy and computational speed are both of very high import in these applications.

### 6.3. Healthcare

Monitoring the health of hospital and nursing home patients is one of the fields in which machine learning has been found to be increasingly useful. The AI models trained for these purposes are varied depending on the exact nature of the task they are created to accomplish [69,70]. Applications involving the monitoring of the status of specific organs of patients can rely on various different medical equipment as well as visual and thermal cameras, such as monitoring a patient’s heart rate or brain activity, which are achieved with electrocardiograms and electroencephalograms. Fast performance of the machine learning models is of even greater importance in these scenarios as they can quite literally be about "life and death". Other health monitoring applications can refer to posture recognition and monitoring systems that rely on motion sensors and cameras to identify the posture of a given patient and inform their caretakers in case of any danger.

### 6.4. Drones

Aerial drones, or unmanned aerial vehicles, have a long history of military use, but have become increasingly utilized in everyday life over the past decade, be it for package delivery, remote video recording, wildlife research, or simply for recreational purposes. Many of these drones are of the quadcopter variety [71]. While most drones require remote piloting, there has been an increasing element of automation to their navigation [72,73], odometry, landing, and trajectory systems. AI models trained for these purposes use pathways, object images, and balance data models. While performance speed is an important factor for these models, accuracy takes far greater precedence as even the slightest misclassification can result in damage to or the destruction of the drone.

### 6.5. Agriculture

Different agricultural sectors have also started making use of machine learning. Object detection and facial recognition models are customized for recognizing individual animals during feeding and drinking to measure their overall consumption as well as monitor animal behavior and health. Object detection machine learning models are also used in farming crops for identifying weeds within the field, damaged crops, and crops ready for harvest, as well as any damage to the field and its fences. In both instances, the detection accuracy and energy consumption of the models are far more important than the performance speed.

## 7. Application Based System Comparison

**Figure 2:**
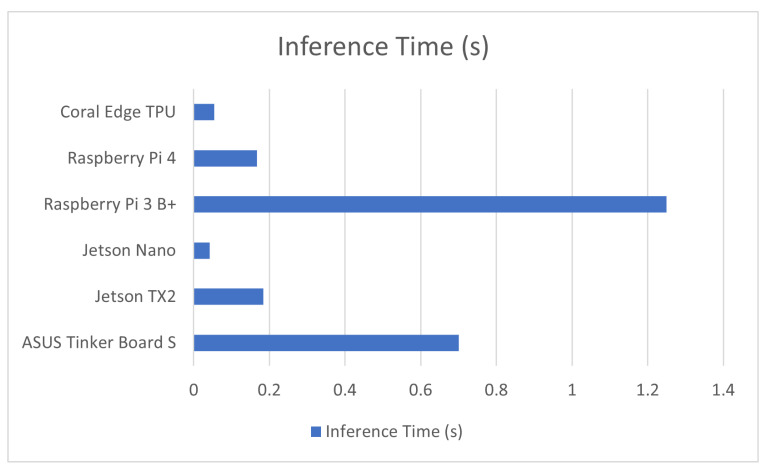
Average inference time in agricultural computer vision for devices used in this application.

**Figure 3:**
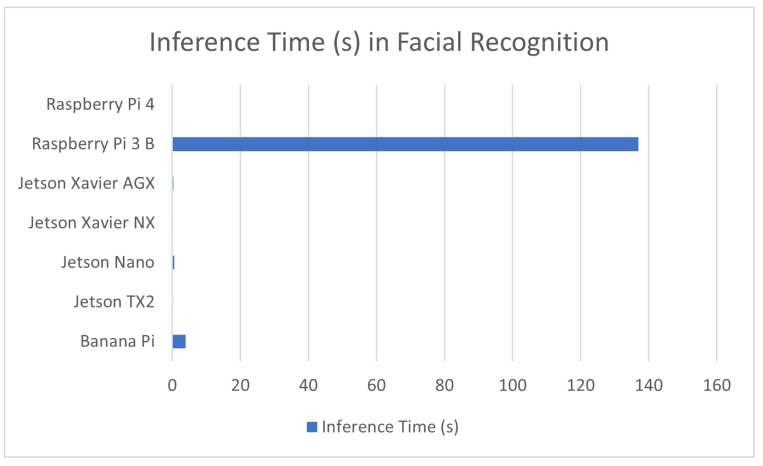
Average Inference time in facial recognition for devices used in this application.

**Figure 4:**
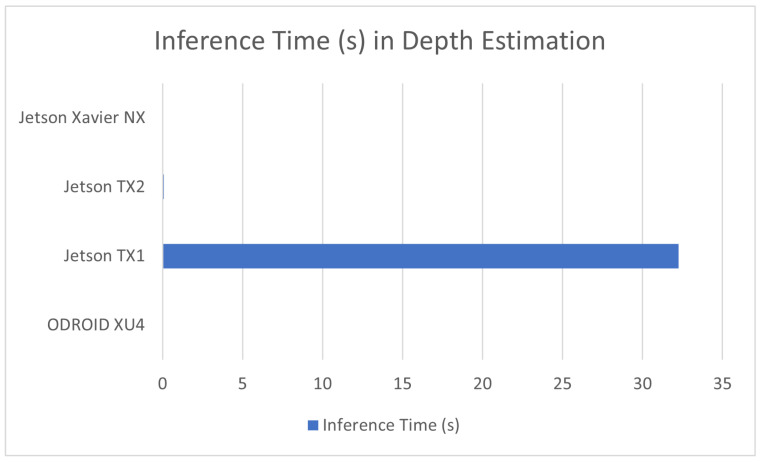
Avg. inference time in depth estimation for devices used in this application.

**Figure 5:**
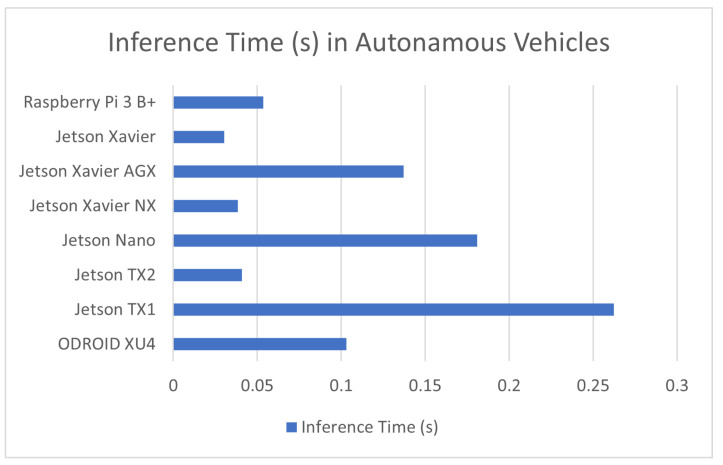
Average inference time in autonomous vehicle obstacle recognition in devices used in this application.

**Figure 6:**
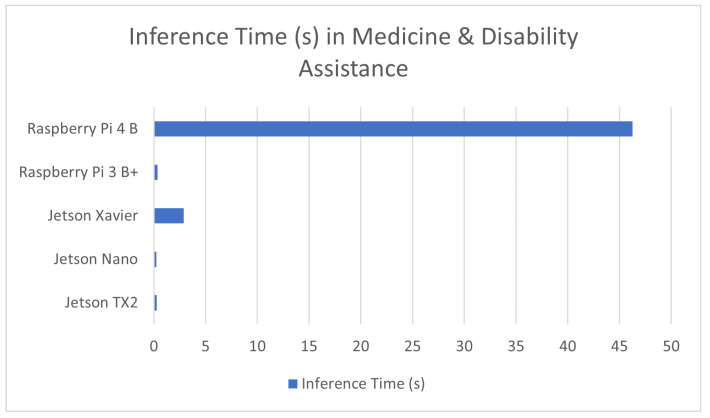
Average inference time in medicine and disability assistance in devices used in these applications.

**Figure 7:**
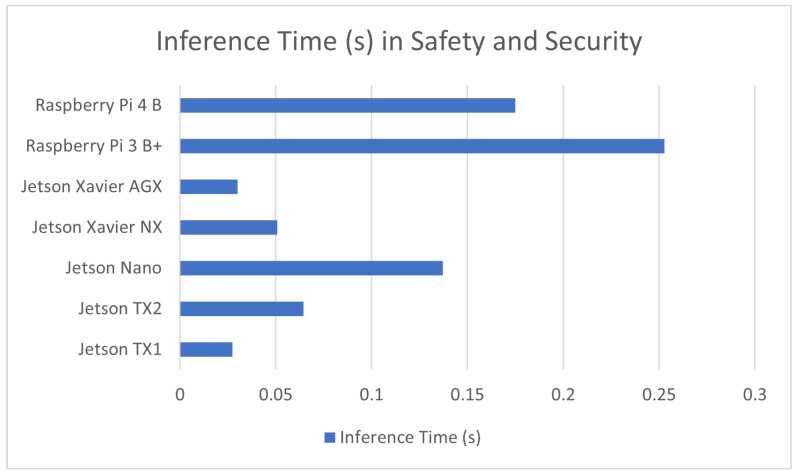
Average inference time in safety and security in devices used in these applications.

**Figure 8:**
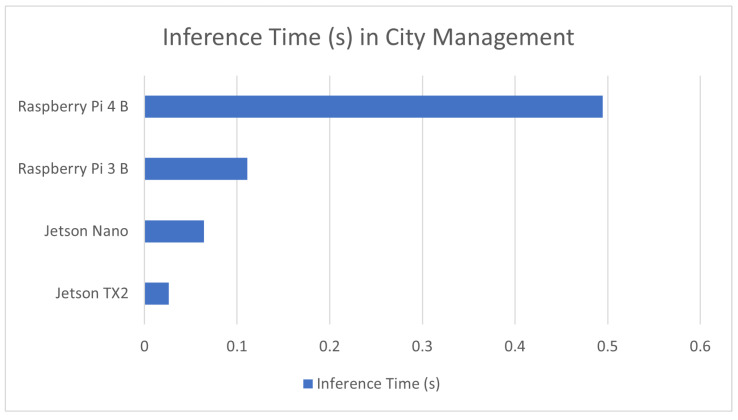
Average inference time in devices used in city management applications.

**Figure 9:**
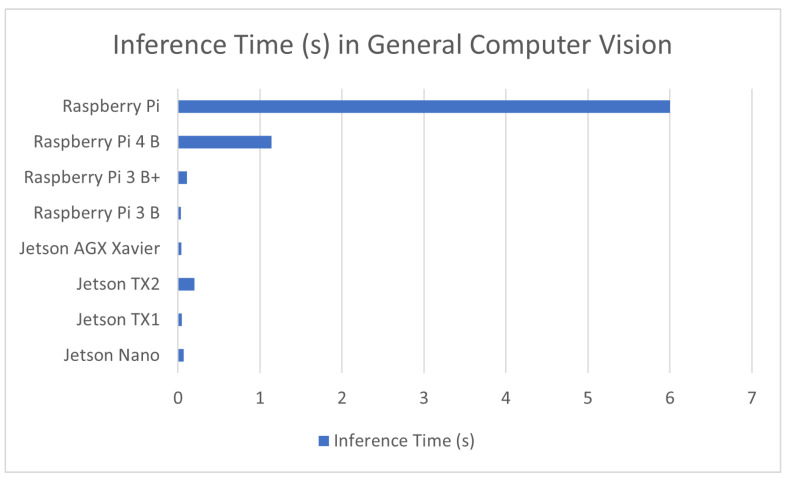
Average inference time in embedded computer vision devices.

As previously discussed, most review work on embedded machine learning has been focused on the implementation of modified ML architecture on specific embedded devices, whereas in this work, our focus is on identifying the advantages certain systems provide for specific applications and sensing schemes. For this purpose, we have divided our sources into the following categories with a summary of each presented in the Table 2, Table 3, Table 4, Table 5, Table 6, Table 7, Table 8, Table 9, Table 10, Table 11, Table 12 after the conclusion section. The systems are then compared by their performance and cost, the former being assessed differently depending on the task for which the machine learning model is trained. The method used for analyzing the performance is different from source to source and heavily dependent on the specific application and sensory system. Each sourced paper used a different method for analyzing model accuracy and inference speed. Alongside the power consumption, the mean of all the final results is used to assess the overall performance of each embedded system and presented in Figure 2, Figure 3, Figure 4, Figure 5, Figure 6, Figure 7, Figure 8 and Figure 9.

### 7.1. Image Recognition, Object Detection, and Computer Vision

As previously stated, different machine learning methods have been seeing an ever-increasing application within various fields, among these methods is the broad field of computer vision, which includes image and object detection. These applications can range from security and agriculture to autonomous vehicles—we have further divided these applications into the specific field in which they are applied.

#### 7.1.1. Crop Identification

As previously discussed, like many other professions, machine learning has been seeing an increasing level of application within the field of crop and animal agriculture. This application can range from smart affordable farming solutions such as in [74] to the monitoring of ripened produce as in [75]. While time is valuable in any discipline, for agricultural machine learning applications, it is not nearly as much of a priority as power consumption and accuracy. Most of the applications covered in this review involve the usage of object recognition algorithms for the detection of various field or crop features but there are other applications that are analyzed as well. The performance of these applications is covered in Table 2 in addition to a comparison graph provided in Figure 2.

**Table 2:** Computer Vision in Agriculture.

Paper Title	Hardware	Application	Sensor	Accuracy	Power Consumption	Inference Time
[76]	ASUS Tinker Board S	Crop identification via aerial drone	Logitech C925e wWebcam	89.44%	8 Watts for both sensor and system	0.7 s
[77]	Google Edge TPU, NVIDIA Jetson TX2	Vineyard Landmark extraction for robot navigation in steep slope vineyard environment through vine trunk identification	Raspberry Pi infrared camera, Mako G-125C infrablue camera	52.98%	15 Watts for both sensor and system	54.20 ms
[78]	Raspberry Pi 3 B+, with and without a neural compute stick, (Intel Movidius) NVIDIA Jetson Nano	Protect crops from ungulate attacks	Camera module (Raspberry Pi)	62.41%	10 Watts for both sensor and system (Jetson) 3.4 Watts for both sensor and system (RaPi)	67.57 ms (Jetson) 1.25 s (RaPi)
[79]	NVIDIA Jetson Nano	Detection of ripe coffee beans	Intel realsense depth camera D435	97.23%	14 Watts for both sensor and system	17.49 ms
[80]	NVIDIA Jetson TX2	Crop recognition for robotic weeding	Canon PowerShot SX150 IS camera	95.9%	12.5 Watts for both sensor and system	8.9 ms
[81]	NVIDIA Jetson TX2	Accurate weed detection for micro aerial vehicles	Multispectral camera	79.9%	15 Watts for both sensor and system	0.56 s
[82]	Raspberry Pi 4	Weed identification for herbicide	Raspberry Pi camera module version 2.0 with an 8-megapixel Sony IMX219 sensor	96%	6.88 Watts for both sensor and system	0.167 s
[83]	NVIDIA Jetson TX2	Loose fruit detection for oil palm	Camera	94%	10 Watts for both sensor and system	Not Stated
[84]	NVIDIA Jetson TX2	Intelligent pest detection	High-resolution optical drone camera	89.72%	7.5 Watts	114.89 ms

#### 7.1.2. Face and Expression Recognition

Facial recognition is one of the most well known applications in the field of computer vision—many personal projects, academic research studies, and computer applications have been developed regarding or using facial recognition. There are also many specialized models based on facial recognition, such as facial recognition models for animals [85], or facial expression recognition models that make use of existing facial recognition technologies as a baseline [86]. The priority in facial recognition models is dependent on the application as models used for security purposes would need to have both high accuracy and inference speed, while commercial application models are not under as much scrutiny. Most of the sources used in this review either implement facial recognition directly [87], or use it as a basis for emotion and personality assessment as well [85]. The performance of these applications is covered in Table 3 in addition to a comparison graph provided in Figure 3.

#### 7.1.3. Depth Estimation

Depth estimation is a sub-field of machine learning that attempts to estimate depth within 2D images. It involves the use of pixel shape and orientation for the identification of the distance of objects within 2D images and video from the device that recorded it. Its utility is mainly in photography and depth estimation for self-driving vehicles, while within our sources, it was mostly used for personal projects such as in [88]. The performance of these applications is covered in Table 4 as well as a comparison graph being provided in Figure 4.

**Table 3:** Computer Vision in Face Recognition.

Paper Title	Hardware	Application	Sensor	Accuracy	Power Consumption	Inference Time
[86]	Banana Pi	Emotion and Personality Recognition	Thermal Camera (Vanadium Oxide Microbolometer with Chalcogenide Lens and a Field of View 36O.)	87.87%	4 Watts for both sensor and system	3.851 s
[89]	Nvidia Jetson Nano, Nvidia Jetson TX2, Nvidia Jetson Xavier NX, Nvidia Jetson Xavier AGX	Facial recognition inference comparison between edge and cloud devices	None	99.63%	5 Watts (Nano) 7.5 Watts (TX2) 10 Watts (Xavier NX & AGX)	0.37 s (Nano) 0.4 s (TX2) 0.18 s (Xavier NX) 0.28 s (AGX)
[2]	NVIDIA Jetson Nano	Analyze face structure from video feed and detect drowsiness from facial features	Webcam camera	83.31%	15 Watts for both sensor and system	2 s
[90]	NVIDIA Jetson Nano	Face mask detection system	TGCAM-2000STAR camera	99.02%	17 Watts for both sensor and system	30.18 ms
[87]	Raspberry Pi 3 model B	Facial biometric scan	Pi camera	97.1%	2.8 Watts for both sensor and system	2.283 min
[91]	Raspberry Pi 4	High-accuracy facial recognition	Webcam	75.26%	14 Watts for both sensor and system	74.15 ms
[92]	Raspberry Pi 4	Facial recognition and facial expression recognition	Logitech c270 camera	98%	14 Watts for both sensor and system	71.14 ms
[93]	NVIDIA Jetson Nano, NVIDIA Jetson TX2	Facial ID for security	Camera	94%	5 Watts (Nano) 7.5 Watts (TX2)	0.1 s (Nano) 33.33 ms (TX2)
[94]	NVIDIA Jetson TX2	Lightweight facial recognition for embedded systems	Camera	58.7%	1.4 Watts	29 ms

**Table 4:** Computer Vision in Depth Estimation.

Paper Title	Hardware	Application	Sensor	Accuracy	Power Consumption	Inference Time
[88]	NVIDIA Jetson TX1	Monocular depth estimation (MDE) (estimating depth from a single image or video frame)	Camera	78.3%	5 Watts	32.26 ms
[95]	ODROID XU4 NVIDIA Jetson TX2	Collision checking for small aerial vehicles navigation	FLIR thermal imaging camera	35.3%	1.5 Watts (ODROID) 7.5 Watts (TX2)	30 ms (ODROID)
[75]	ODROID XU4	Computationally inexpensive misclassification minimization for aerial vehicles	D435i Depth Camera	45.8%	1.5 Watts 4.9 Watts for System and Sensor	36.46 ms
[96]	NVIDIA Jetson Xavier NX	Depth estimation	Monocular camera	87.8%	10 Watts	0.03 s
[97]	NVIDIA Jetson TX2	Personal fall detection system	Image depth camera, RGB camera	98%	7.5 Watts	66.67 ms

#### 7.1.4. Autonomous Vehicle Obstacle Recognition

One of the most widespread and focused implementations of machine learning, specifically, embedded machine learning, is in autonomous or assisted vehicles. Self-driving cars have been a staple of both science fiction and practical research for decades, but in the past decade, they have come increasingly close to reality. Advances in machine learning have been one of, if not the largest, driving factors behind this. While there are many different aspects of driving that a machine-earning algorithm could automate, from speed adjustment to the piloting of the vehicle in different directions, the focus in this review is mainly on the implementations of detection schemes for the various obstacles a vehicle can encounter, from other cars to pedestrians [98], road signs [99], traffic lights [5], and speed bumpers [11]. Due to the extremely dangerous nature of this application, systems used for these implementations need to be both as accurate and as fast as possible. The performance of these applications is covered in Table 5 in addition to a comparison graph provided in Figure 5.

**Table 5:** Computer Vision in Autonomous vehicles.

Paper Title	Hardware	Application	Sensor	Accuracy	Power Consumption	Inference Time
[98]	ODROID XU4 NVIDIA Jetson Xavier	Nighttime pedestrian detection systems for cars	FLIR A325sc thermal camera	75.7%	1.5 Watts (ODROID) 10 Watts (Xavier)	103 ms (ODROID) 43.3 ms (Xavier)
[5]	NVIDIA Jetson TX1, NVIDIA Jetson TX2	Lightweight real-time traffic light detection for autonomous vehicles	AVT camera (only used for data collection)	99.3%	5 Watts (TX1) 7.5 Watts (TX2)	83.3 ms (TX1) 71.4 ms (TX2)
[1]	NVIDIA Jetson TX2	Road marking detection for autonomous vehicles	Camera	96.9%	7.5 Watts	47 ms
[100]	NVIDIA Jetson TX2	Lightweight road object detection for autonomous vehicles	Camera	80.39%	7.5 Watts	31 ms
[101]	NVIDIA Jetson Xavier	Lightweight Multitask object detection and semantic segmentation for autonomous vehicles	N/A	98.31%	10 Watts	17.36 ms
[102]	NVIDIA Jetson Xavier NX	Path Planning for self-driving vehicles and robotic systems	Camera	93%	10 Watts	48.57 ms
[103]	NVIDIA Jetson Nano	Thermal object detection for assisted driving	LWIR prototype thermal camera	86.6%	5 Watts	333.33 ms
[104]	NVIDIA Jetson Xavier NX	Road obstacle detection for vehicles	20 Hz stereo camera	98.1%	10 Watts	28.23 ms
[99]	NVIDIA Jetson TX1	Traffic sign identification for smart vehicles	USB webcam	96%	5 Watts	670 ms
[105]	NVIDIA Jetson AGX Xavier	Object detection and recognition and energy management for autonomous vehicles	N/A (can theoretically use onboard camera or radar)	99.63%	10 Watts	260 ms
[106]	Raspberry Pi 3 Model B+	Scalable and computationally cheap networks for autonomous driving	Raspberry Pi camera	97.75%	2.1 Watts	3 ms
[11]	Raspberry Pi 3 Model B+	Speed bump detection for autonomous vehicles	Raspberry Pi camera	97.89%	2.1 Watts	104 ms
[107]	NVIDIA Jetson Nano	Algorithm review for self-driving car navigation	Mini camera IMX-219	80.5%	5 Watts	Not Stated
[9]	NVIDIA Jetson TX1	Real-time pedestrian detection for autonomous vehicles	Zed Stereo camera	88.44%	5 Watts	33.3 ms
[108]	NVIDIA Jetson TX2	Real-time vehicle detection on embedded systems	N/A	85.6%	7.5 Watts	59.52 ms
[109]	NVIDIA Jetson AGX Xavier	Uncertainty-based real-time object detection for autonomous vehicles	Camera	68.7%	10 Watts	14.35 ms

#### 7.1.5. Computer Vision in Medical Diagnosis and Disability Assistance

An interesting and beneficial application of computer vision is its use in the diagnosis of medical conditions and in assisting individuals with disabilities. Many of the sources presented in this review made use of RGB and thermal imaging of patients to perform object detection and image classification to find any signs of medical conditions such as melanoma [110] or diabetes [111], while others presented systems for assisting the visually impaired [112]. In both presented fields of application, while a very high accuracy is of extreme importance, a high inference speed is also paramount to any aides to special needs individuals. The result of these benchmarks is covered in Table 6 in addition to a comparison graph provided in Figure 6.

#### 7.1.6. Computer Vision in Safety and Security

A more novel application of Computer vision models is its use in security systems as well as safety oversight networks. The sources presented in this section cover applications in detecting violent assaults [12] and mining personnel safety [3] to detecting survivors of severe natural disasters [113]. Most of these applications make use of RGB video and image cameras to perform detection and recognition. The result of these benchmarks is covered in Table 7 in addition to a comparison graph provided in Figure 7.

**Table 6:** Computer Vision in Medical and Special Aide Applications.

Paper Title	Hardware	Application	Sensor	Accuracy	Power Consumption	Inference Time
[112]	NVIDIA Jetson TX2	Visual aid system for the blind via real-time object detection	Webcam	99.82%	7.5 Watts	Not Stated
[114]	NVIDIA Jetson TX2	Localize veins from color skin images.	2-CCD multi-spectral prism camera (JAI AD-080-CL)	78.27%	7.5 Watts	530 ms
[115]	Raspberry Pi 4, NVIDIA Jetson Xavier	COVID Identification through chest CT scans	CT Scanner	98.8%	4 Watts (Pi 4) 10 Watts (Xavier)	23.3 s (Pi 4) 2.9 s (Xavier)
[116]	NVIDIA Jetson Nano	Posture recognition system for medical surveillance	RGB camera	83%	5 Watts	476 ms
[117]	NVIDIA Jetson TX2	Diabetes diagnosis	Jetson TX2 onboard camera	91.8%	7.5 Watts	48 ms
[118]	Raspberry Pi 3 Model B+	Reading assistance for blind people	Raspberry Pi camera module V2	100%	2.1 Watts	1 s
[110]	Raspberry Pi 3 Model B+	Early skin cancer detection	IR camera	98%	2.1 Watts	62 ms
[119]	Raspberry Pi	Cervical cancer prevention	PiCamera	90%	Not Stated	5.2 s
[120]	Raspberry Pi 4 Model B	Dog health monitoring through posture analysis	Smart camera network	100%	4 Watts	69.24 s
[111]	NVIDIA Jetson Nano	Diabetic ulcer detection	Thermal Camera	97.9%	5 Watts	Unspecified
[121]	NVIDIA Jetson Xavier NX	Colonoscopy	Colonoscopy camera	100%	10 Watts	Unspecified
[122]	NVIDIA Jetson Nano	Travel assistance for the visually impaired	Optical RGB camera	94.87%	5 Watts	22.22 ms
[123]	Raspberry Pi 3 Model B+	Activity recognition for medical monitoring and rehab	Wearable Sensor	96.63%	2.1 Watts	167.773 ms

**Table 7:** Computer Vision in Safety and Security Applications.

Paper Title	Hardware	Application	Sensor	Accuracy	Power Consumption	Inference Time
[124]	Raspberry Pi	Sign language recognition	Thermal camera	99.52%	Not Stated	30 ms
[125]	NVIDIA Jetson Xavier NX	Proposal of a fast and accurate method of power line edge intelligent inspection	UAV camera	55.6%	10 Watts	3.5 ms
[3]	NVIDIA Jetson TX1	Production safety oversight in coal mines	Video Surveillance camera	76.7%	5 Watts	27.25 ms
[126]	NVIDIA Jetson Nano	Passenger safety monitoring	360◦ view camera	85%	5 Watts	Not Stated
[127]	NVIDIA Jetson TX2, NVIDIA Jetson Nano	Hard hat detection on construction site	Surveillance camera	97.14%	7.5 Watts (TX2) 5 Watts (Nano)	68.03 ms (TX2) 111 ms (Nano)
[128]	NVIDIA Jetson TX2	Detecting and tracking sinkholes via video streaming	Video camera	90.61%	7.5 Watts	17 ms
[129]	NVIDIA Jetson TX2	Concrete damage detection on the surface of buildings	Logitech Camera	94.24%	7.5 Watts	33 ms
[130]	NVIDIA Jetson AGX Xavier	Railway defect detection	Camera	93.5%	10 Watts	29.94 ms
[131]	Raspberry Pi 4 Model B	Biometric scan for entry control	Raspberry Pi NoIR camera	97.2%	4 Watts	Not Stated
[132]	Raspberry Pi 4	Real-time fire detection	Camera	97.5%	4 Watts	100 ms
[12]	Raspberry Pi 4	Violent assault recognition	Surveillance camera (no actual live testing)	92.05%	4 Watts	250 ms
[133]	Raspberry Pi 3 Model B+, Intel Neural Compute Stick 2	Security surveillance	Surveillance camera	94%	2.1 Watts	5.5 ms
[134]	NVIDIA Jetson Nano	Security surveillance for abnormal activity detection	Logitech C270 Camera	89%	5 Watts	250 ms
[135]	NVIDIA Jetson Nano	Security surveillance for unusual behavior	HD camera	97.5%	5 Watts	Not Stated
[136]	NVIDIA Jetson Xavier NX	Fire and smoke detection	Camera	100%	10 Watts	100 ms
[137]	NVIDIA Jetson TX2	Monitoring vehicle driver tiredness in real time	Infrared Camera	94%	7.5 Watts	45.45 ms
[138]	NVIDIA Jetson TX2	Real-time security surveillance for acts of violence	RaspiCam camera, panoramic spherical camera	Not Stated	7.5 Watts	185 ms
[139]	NVIDIA Jetson Nano, Raspberry Pi 3 Model B+	Rescue operation robot computer vision	No IR filter camera, LiDAR, Raspi Cam NOIR V2.1	78.6%	7.5 Watts (Nano) 2.1 Watts (Pi 3)	50 ms (Nano) 500 ms (Pi 3)
[140]	Raspberry Pi	CPU heat tracking	Infrared thermal sensor	90.72%	Not Stated	12.3 ms
[141]	NVIDIA Jetson Xavier NX	Real-time image processing for fusion diagnostics	Thermal image camera	Not Stated	10 Watts	48.97 ms
[142]	NVIDIA Jetson Nano	Automobile fog lamp intelligent control	IMX219 camera	97.5%	5 Watts	Not Stated
[113]	NVIDIA Jetson TX2	Rescue of natural disaster survivors through drone object detection	Zenmuse XT2 gimbal camera	61.97%	7.5 Watts	37.6 ms
[143]	NVIDIA Jetson Nano	Power system cyber security	N/A	99.96%	5 Watts	Not Stated

#### 7.1.7. Smart City Management

Smart cities are an increasingly used term within tech circles that refers to, among other things, the usage of machine learning and AI for the automation of many aspects of city management. Many of these applications are related to traffic management [14] or to the profiling of individuals [144]. It is very important for these models to be able to handle a large number of objects at any given time; for this reason, inference time is of a higher priority for these applications. Most of these applications make use of RGB video cameras to perform detection and recognition. The result of these benchmarks is covered in Table 8 as well as a comparison graph being provided in Figure 8.

#### 7.1.8. General Embedded Computer Vision

Many of the sources presented in this review could not fit into a large enough application category of their own. These sources ranged from works that were focused on the visual location of robotic limb grasping points [145] to ones studying the identification of individuals via their clothing [146]. For that purpose, these sources were all included within a generalized category presented in Table 9 as well as the comparison graphs shown in Figure 9.

**Table 8:** Computer Vision in City Management.

Paper Title	Hardware	Application	Sensor	Accuracy	Power Consumption	Inference Time
[14]	NVIDIA Jetson TX2	Traffic flow detection and management	Canon EOS550D camera	92%	7.5 Watts	26.39 ms
[147]	NVIDIA Jetson Nano	Real-time metro passenger volume enumeration	HD video recording camera	97.1%	5 Watts	128.2 ms
[148]	Raspberry Pi 4 Model B	Smart Urban waste management	Pi Camera	91.76%	4 Watts	358.9598 ms
[149]	Raspberry Pi 4 Model B	Garbage identification for recycling	Camera	92.62%	4 Watts	630 ms
[144]	Raspberry Pi 3 Model B	Pedestrian profile recognition	FLIR Lepton thermal camera	74.63%	1.4 Watts	111 ms
[150]	NVIDIA Jetson Nano	Car counter Traffic management	Logitech c922 webcam	Not Stated	5 Watts	Not Stated
[151]	NVIDIA Jetson Nano	Smart city traffic management	Camera	90%	5 Watts	25 ms
[152]	NVIDIA Jetson Nano	Visual garbage detection	N/A (most likely a video camera)	94.56%	5 Watts	40 ms
[153]	NVIDIA Jetson Nano	AI traffic light control	Raspberry Pi camera	90%	5 Watts	Not Stated

**Table 9:** General Embedded Computer Vision.

Paper Title	Hardware	Application	Sensor	Accuracy	Power Consumption	Inference Time
[146]	NVIDIA Jetson AGX Xavier	Person detection using top clothing	N/A	92.57%	10 Watts	41.67 ms
[154]	NVIDIA Jetson TX1	Detecting, tracking, and geolocating based on a monocular camera of an aerial drone	Monocular Camera	97.6%	5 Watts	75.76 ms
[155]	NVIDIA Jetson TX2	Drone detection	Spherical Camera (Ricoh Theta S)	88.9%	5 Watts	33.33 ms
[156]	NVIDIA Jetson TX2	Resource-constrained object tracking	N/A	55%	7.5 Watts	72.89 ms
[157]	NVIDIA Jetson TX2	Object detection and object tracking on drones with limited power and computational resources	Logitech BRIO camera	90%	7.5 Watts	243.9 ms
[145]	NVIDIA Jetson Nano	Identifying and detecting suitable grasping point on objects for robotic limbs	A Basler acA2500-14uc industrial RGB camera with Computer M3514-MP lens	Not Stated	5 Watts	48 ms
[158]	NVIDIA Jetson TX2	Navigation for indoor autonomous drones	Fisheye lens on the PointGrey Firefly camera	75.5%	7.5 Watts	34.54 ms
[159]	NVIDIA Jetson TX2, NVIDIA Jetson Nano	Object detection via template tracking	N/A	Not Stated	7.5 Watts (TX2) 5 Watts (Nano)	Not Stated
[160]	NVIDIA Jetson TX2	Target tracking amongst static and dynamic obstacles	Drone camera	Not Stated	7.5 Watts	Not Stated
[161]	NVIDIA Jetson TX2	Underwater object gripping point detection	ZED binocular camera	Not Stated	7.5 Watts	90.09 ms
[162]	NVIDIA Jetson TX2	Intelligent weapon targeting system	N/A	68.9%	7.5 Watts	60 ms
[163]	NVIDIA Jetson AGX Xavier	Object recognition for unmanned surface vehicles	High-definition photoelectric vision sensor	81.74%	10 Watts	37.36 ms
[164]	Raspberry Pi 3 Model B+	Drone landing automation	Raspberry Pi v1.3 camera with a fisheye lens	Not Stated	2.1 Watts	37.36 ms
[10]	Raspberry Pi 3 model B	Image recognition for sea life	Pi Camera v2.1	89.81%	1.4 Watts	33.33 ms
[165]	Raspberry Pi 3 Model B+	Image classification	N/A	83.7%	2.1 Watts	180 ms
[166]	Raspberry Pi	Counting individuals within a given video feed	Camera	90%	1.4 Watts	Not Stated
[167]	Raspberry Pi	Fish recognition for underwater drones	360 degrees panoramic camera	87%	1.4 Watts	6 s
[168]	NVIDIA Jetson Nano	Identifying different plant species	Photo camera	97.5%	5 Watts	Not Stated
[169]	Nvidia Jetson Nano, Nvidia Jetson TX1, Raspberry Pi 4	Artistic photography aesthetic score prediction	N/A	91.02%	5 Watts (Nano and TX1) 4 Watts (Pi 4)	37 ms (Nano) 17.9 ms (TX1) 1.14 s (Pi 4)
[170]	NVIDIA Jetson Nano	Underwater object detection	N/A (visual camera in case of field testing)	74.77%	5 Watts	125 ms

### 7.2. Non-Vision-Related Machine Learning

Among the sources used for this review, a number were unrelated to any sub-field of computer vision and relied on different sensing schemes from LiDar [171] to ultrasound [13] for gathering training data and implementation, in applications from waste management [148] to heart monitoring [13]. While the sensing scheme and overall application of these models vastly differed from one another, their numbers for each application and sensor were not sufficient for a proper basis-by-basis comparison. For this reason, they are displayed within Table 10.

### 7.3. Embedded Machine Learning Optimization

**Figure 10:**
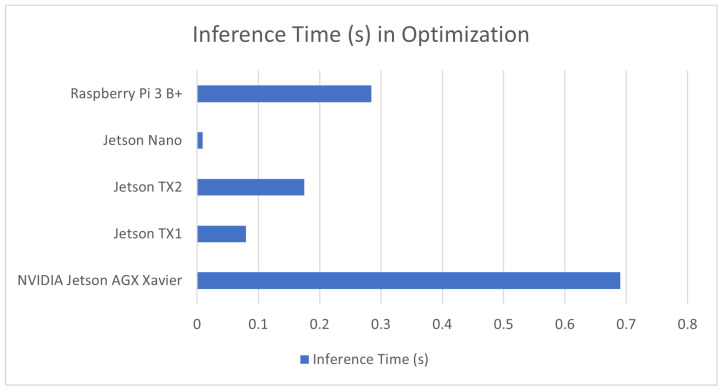
Average inference time in devices used for testing model optimization methods.

Some of the sources in this review did not look into new applications of machine learning, but rather sought to optimize the performance of existing machine learning architecture on embedded system devices. The optimizations ranged from improving the effectiveness of image captioning models on the NVIDIA Jetosn TX2 [172] to pruning deep neural nets [173]. It should be noted that unlike the other sources in this review, most of these papers did not have sensing schemes. The result of these benchmarks is covered in Table 11 in addition to a comparison graph provided in Figure 10.

### 7.4. Benchmarks, Reviews, and Machine Learning Enhancements

**Figure 11:**
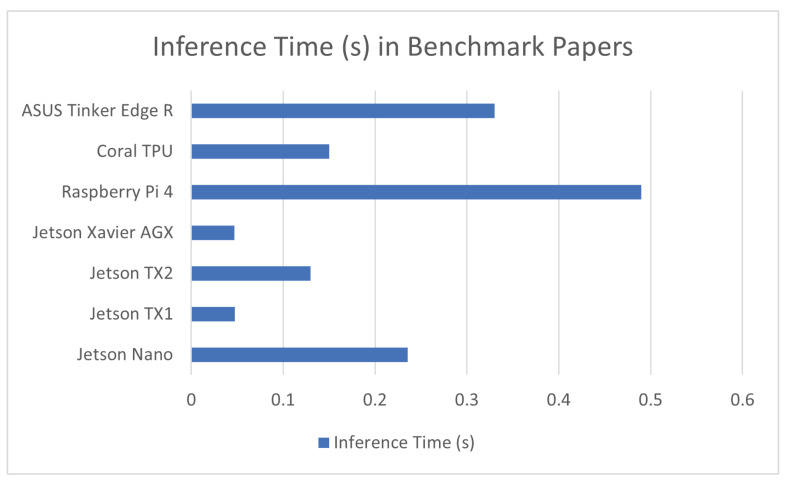
Average inference time in devices covered in referenced benchmark papers.

Among the sources used for this review, there were works of research that were not focused on the introduction of a specific application or a new method for the implementation of machine learning tasks for any field. These papers either attempted to perform benchmarks of different embedded system hardware via the implementation of specific machine learning architectures on them [20] or tried to augment the learning rate of machine learning models and implement their work on embedded computing systems [23]. While most of the work that fell into this category did not include any sensing schemes, the data gathered in them were highly relevant to this work and were for that reason included in this review. The result of these benchmarks are covered in Table 12 and a comparison graph is provided in Figure 11.

**Table 10:** LiDar, Radar, Audio, and Motion Recognition Models.

Paper Title	Hardware	Application	Sensor	Accuracy	Power Consumption	Inference Time
[13]	NVIDIA Jetson Nano, Raspberry Pi 3	Early cardiovascular disease prevention through ultrasound	Ultrasound	90.7 %	5 Watts (Nano) 1.4 Watts (Pi 3)	2.78 ms (Nano) 6.95 ms (Pi 3)
[174]	Raspberry Pi 3	Patient anesthesia monitoring	Electroencephalogram	95%	1.4 Watts	20 ms
[175]	Raspberry Pi 3	Human posture detection	Wireless body sensors (motion sensors, inertial sensors)	98.28%	1.4 Watts	20 ms
[176]	NVIDIA Jetson Nano	Epileptic seizure detection	Electrocardiogram	91.58%	5 Watts	Not Stated
[177]	NVIDIA Jetson TX2	Low-power multimodal data classification	Stand-alone dual-mode Tongue Drive System	98%	7.5 Watts	1.6 ms
[178]	Raspberry Pi Model 3	Driver behavior monitoring	IMU sensor, Shimmer Version 3 wearable body sensors	73.02%	1.4 Watts	4.357 s
[179]	Raspberry Pi 3 Model B+	Smart Urban waste management	Ultrasonic sensor	88.43%	2.1 Watts	960 ms
[180]	Raspberry Pi 3 Model B	Fault detection in AC electrical systems	Photoelectric sensor	99.37%	1.4 Watts	31 ms
[181]	Raspberry Pi 3 Model B+	Target classification at road gates with radar SVM	Radar	Not Stated	2.1 Watts	Not Stated
[182]	Raspberry Pi 3 Model B+	Human activity recognition	Wearable multimodal sensors	99.21%	2.1 Watts	153 ms
[183]	Raspberry Pi 3B+	Speech recognition	Audio sensor	96.82%	2.1 Watts	270 ms
[4]	Raspberry Pi 3B, NVIDIA Jetson TX1, NVIDIA Jetson TX2	Psychological stress monitoring	Heart rate and accelerometer sensors	96.7%	1.4 Watts (Pi 3) 5 Watts (TX1) 7.5 Watts (TX2)	189 ms (Pi 3) 2.8 ms (TX1) 4.7 ms (TX2)
[184]	Raspberry Pi 3 Model B	Motor fault diagnosis	Hall effect sensor	97.05%	1.4 Watts	3.4 s
[185]	Raspberry Pi 4 Model B	Machine state monitoring	Vibration Sensor, Accelerometers	98%	4 Watts	1.002 s
[186]	Raspberry Pi	Asthma risk prediction	SDS011 air quality sensor	99%	1.4 Watts	Not Stated
[8]	Raspberry Pi 3 Model B	Speech source identification	SSL sensors, microphones	89.68%	4 Watts	21 ms
[187]	NVIDIA Jetson Nano	Battery charge management	GY169 current converter sensor module	RMSE of 1.976	5 Watts	Not Stated
[188]	NVIDIA Jetson TX2	Food quality analysis	Nuclear magnetic resonance spectrometer, infrared spectrometer	95%	7.5 Watts	4 ms
[189]	NVIDIA Jetson Nano	Pot plant species identification and watering needs monitoring	Capacitive Soil Moisture sensor, Water Level Sensor	Not Stated	5 Watts	Not Stated
[190]	NVIDIA Jetson Nano	Radio frequency ID recognition	Universal software radio peripheral	89.27%	5 Watts	18 min
[171]	NVIDIA Jetson Xavier NX	Trajectory tracking for small drones	Velodyne Lite 16 Lidar sensor	83%	10 Watts	100 ms

**Table 11:** Embedded Machine Learning Optimization Papers.

Paper Title	Hardware	Application	Sensor	Accuracy	Power Consumption	Inference Time
[172]	NVIDIA Jetson TX2	Improve the effectiveness of Image Captioning	N/A	65.7%	7.5 Watts	230 ms
[191]	NVIDIA Jetson TX2, NVIDIA Jetson Nano	Latency estimation on embedded systems	N/A	96.39 % (Nano) 95.82 % (TX2))	5 Watts (Nano) 7.5 Watts (TX2)	13.74 ms (Nano) 6.7 ms (TX2)
[192]	NVIDIA Jetson Nano	Real-time video analysis for edge computing	Video camera	85%	5 Watts	11.21 ms
[193]	NVIDIA Jetson TX2	Low-power and real-time deep learning-based multiple object visual tracking	5MP CSI camera	N/A	7.5 Watts	100 ms
[173]	NVIDIA Jetson TX2	Filter Pruning DNNs	N/A	93.51%	7.5 Watts	8.01 ms
[194]	NVIDIA Jetson AGX Xavier	Energy-efficient acceleration of deep neural networks	N/A	N/A	10 Watts	Not Stated
[195]	NVIDIA Jetson TX1	Semantic Segmentation for autonomous vehicles	N/A	87.3%	5 Watts	24 ms
[196]	NVIDIA Jetson TX2	Improve semantic segmentation performance in contexts of various sizes and types in diverse environments	N/A	92.74%	7.5 Watts	92.46 ms
[197]	NVIDIA Jetson TX2, Edge tensor processing unit, neural compute stick, and neural compute stick2	Fusion Pruning DNNs	N/A	90.66%	7.5 Watts	4.7 ms
[198]	NVIDIA Jetson TX2	Reduce computational complexity and memory consumption of CNNs architecture on low-power devices	N/A	93%	7.5 Watts	66.14 ms
[199]	NVIDIA Jetson TX2	Reduce computational complexity and memory consumption of CNNs architecture on low-power devices	N/A	99.3%	7.5 Watts	894.85 ms
[200]	NVIDIA Jetson AGX Xavier	Improve embedded system performance in autonomous vehicles	N/A	98.3%	10 Watts	690 ms
[201]	NVIDIA Jetson TX1	Provide a less resource costly object detection model for embedded systems	N/A	65.7%	5 Watts	135.2 ms
[202]	NVIDIA Jetson Nano	Efficient video understanding	Video camera	74.1%	5 Watts	13.51 ms
[106]	Raspberry Pi 3 Model B+	Scalable and computationally cheap networks for autonomous driving	Raspberry Pi camera	75.78%	5 Watts	284 ms

**Table 12:** Benchmark and Review Papers.

Paper Title	Hardware	Application	Sensor	Accuracy	Power Consumption	Inference Time
[23]	NVIDIA Jetson Nano, Coral Edge TPU, custom convolutional neural network accelerator	Enhance learning rate for ML model with smaller training datasets	N/A (Benchmark paper)	49.6% (Nano) 49.8% (TPU)	5 Watts (Nano) 2 Watts (TPU)	0.3294 s (Nano) 19.8 ms (TPU)
[20]	NVIDIA Jetson Nano, NVIDIA Jetson AGX Xavier	Benchmark analysis of 3d object detection	USB attached video camera (Benchmark paper)	70%	5 Watts (Nano) 10 Watts (AGX)	0.56 s (Nano) 47.61 ms (AGX)
[18]	NVIDIA Jetson Nano, NVIDIA Jetson TX2, Raspberry PI 4	Performance analysis of different hardware for object detection CNNs	N/A (Benchmark paper)	93.8 % (Nano) 93.9% (TX2) 91.6% (Pi)	5 Watts (Nano) 7.5 Watts (TX2) 4 Watts (Pi)	58 s (Nano) 32 s (TX2) 372 s (Pi)
[19]	NVIDIA Jetson TX1	Analysis of DNN architecture in image recognition	N/A (Benchmark paper)	69.52%	5 Watts	10.55 ms
[15]	Asus Tinker Edge R, Raspberry Pi 4, Google Coral Dev Board, NVIDIA Jetson Nano	Presentation and comparison of the performance of the presented systems in terms of inference time and power consumption	N/A (Benchmark paper)	92.5%	4.75 Watts (Tinker) 2.75 Watts (Coral) 2.1 Watts (Pi) 0.9 Watts (Nano)	0.33 s (Tinker) 0.28 s (Coral) 0.21 s (Pi) 0.137 s (Nano)
[22]	Raspberry Pi 4	Space exploration landing site selection	N/A (dataset acquired from images taken by the Mars HiRISE camera)	95%	4 Watts	89 ms
[21]	NVIDIA Jetson Nano, NVIDIA Jetson TX1, NVIDIA Jetson AGX Xavier	Benchmarking paper	N/A	Accuracy Rates Not Stated	5 Watts (Nano & TX1) 10 Watts (AGX)	94 ms (Nano) 84 ms (TX1) 46 ms (AGX)
[17]	NVIDIA Jetson TX2, NVIDIA Jetson Xavier NX, and NVIDIA Jetson AGX Xavier	Benchmarking NVIDIA Jetson systems for visual odometry of flying drones	N/A	Accuracy Rates Not Stated	7.5 Watts (TX2) 10 Watts (NX & AGX)	Speed Rates Not Stated

## 8. Conclusions

Rapid advances have been made in the field of machine learning, causing an explosion of model variety, application, and performance. While many of these models are implemented on powerful stationary computer devices, there are many applications that are faced with cost, power, and size limitations for the specific usage of their models. For this reason, the field of embedded machine learning, which is the implementation of machine learning on embedded computing systems, has also faced a great deal of attention recently. The main challenges faced in embedded machine learning are caused by the severe limitations of embedded system devices in terms of computational performance and power, with different devices having different performances, power requirements, and purchasing costs. In this review, a large collection of research work and implementation of embedded machine learning on Raspberry Pi, NVIDIA Jetson, and a few other series of devices is presented alongside the overall power consumption, inference time, and accuracy of these implementations. In addition, unlike many other reviews of this topic, this paper also includes a presentation of the overall sensing scheme present in many of the works. It was believed that this was a major dimension of embedded machine learning study overlooked by most other reviews on the subject matter. The hope of this review is to familiarize interested researchers in the field of embedded machine learning by giving them a general introduction to it.

Overall, this study contained studies of several generations of embedded systems, specifically, the Nvidia Jetson and Raspberry Pi systems, showing that much like dedicated computing systems, embedded devices have been experiencing steady improvements in the fields of performance and power consumption. More recent Jetson boards such as the TX2 have a far higher performance rate compared to the TX1 while having the same power consumption levels. As these advances continue, it stands to reason that embedded machine learning will see even greater attention and become even more widespread. All of the systems discussed in this work have their own distinct advantages and disadvantages that users would need to consider when choosing a system for their embedded machine learning application. More robust systems with high performance and relatively efficient power usage such as the Jetson Board and Coral Dev Board line tend to be more monetarily expensive, while more affordable options such as the Raspberry and Banana Pi boards tend to have far lower performances. More remote applications such as agricultural object detection systems might need a greater number of low-power systems while not having much emphasis on performance, while autonomous vehicle applications would have a far greater emphasis on performance and accuracy than on cost and power usage. A general table of all sources’ hardware, application, ML architecture, sensor is provided in Table 13 for interested readers.

## Figures and Tables

**Table 1:** Hardware specifications.

Hardware	Processor	RAM	Storage	Power	Maker
ASUS Tinker Board S	Rockchip Quad-Core RK3288 Processor	2 GB Dual-Channel DDR3 Memory	16 GB eMMC Onboard Storage	5 W	Asus
Banana Pi BPI-M2+	H3 Quad-core Cortex-A7 H.265/HEVC 4K	1 GB DDR3 Memory	8 GB eMMC Onboard Storage	5 W	Shenzhen SINOVOIP Co.
Coral TPU Dev Board	NXP i.MX 8M Quad-core Cortex-A53	1 GB LPDDR4 Memory	8 GB eMMC Onboard Storage	(6–10) W	Coral
ODROID-XU4 Board	Exynos5422 Cortex-A15 2 Ghz, Cortex™-A7 Octa core	2 GB LPDDR3 Memory	Flash Storage Interface	15 W	Hardkernel Co.
ASUS Tinker Edge R	Cortex-A72, Cortex-A53, Mali-T860	4 GB LPDDR4 Memory	16 GB eMMC Onboard Storage	65 W	ASUS
NVIDIA Jetson Nano	ARM Cortex-A57 MPCore	4 GB 64-bit LPDDR4	16 GB eMMC 5.1 Onboard Storage	(5–10) W	NVIDIA
NVIDIA Jetson TX1	4 Core ARM Cortex-A57 MPCore	4 GB 64-bit LPDDR4	16 GB eMMC 5.1 Onboard Storage	15 W	NVIDIA
NVIDIA Jetson TX2	6 Core ARM Cortex-A57 MPCore	8 GB 64-bit LPDDR4	16 GB eMMC 5.1 Onboard Storage	25 W	NVIDIA
NVIDIA Jetson AGX Xavier	8 Core ARM v8.2 64-bit MPCore	16 GB 256-Bit LPDDR4x	32 GB eMMC 5.1 Onboard Storage	(10–30) W	NVIDIA
NVIDIA Jetson Xavier NX	6 Core NVIDIA Carmel ARM v8.2 64-bit MPCore	8 GB 128-bit LPDDR4x	microSD storage interface	10 W	NVIDIA
Raspberry Pi 3 Model B	1.2 GHz Broadcom BCM2837 (64 Bit)	1 GB LPDDR2	microSD storage interface	(1.3–1.4) W	Raspberry Pi Foundation
Raspberry Pi 3 Model B+	1.2 GHz Quad-Core ARM Cortex-A53 (64 Bit)	1 GB LPDDR2	microSD storage interface	(1.9–2.1) W	Raspberry Pi Foundation
Raspberry Pi 4 Model B	1.2 GHz Quad-Core ARM Cortex-A72 (64 Bit)	(1/2/4) GB LPDDR2	microSD storage interface	(3.8–4) W	Raspberry Pi Foundation

**Table 13:** Hardware specifications.

Paper Title	Hardware	Application	ML Architecture	Sensor
[76]	ASUS Tinker Board S	Crop identification via aerial drone	SegNet, FCN-AlexNet	Logitech C925e webcam
[86]	Banana Pi	Emotion and Personality Recognition	Hidden Markov Model	Thermal Camera (Vanadium Oxide Microbolometer with Chalcogenide Lens and a Field of View 36O.)
[23]	NVIDIA Jetson Nano, Coral Edge TPU, custom convolutional neural network accelerator	Enhance learning rate for ML model with smaller training datasets	Siamese Neural Network	N/A
[88]	NVIDIA Jetson TX1	Monocular depth estimation (MDE) (estimating depth from a single image or video frame)	Separable Pyramidal pooling Encoder-Decoder (Custom Architecture)	Camera
[77]	Google Edge TPU, NVIDIA Jetson TX2	Vineyard Landmark extraction for robot navigation in steep slope vineyard environment through vine trunk identification	MobileNet V1, MobileNet V2	Raspberry Pi infrared camera, Mako G-125C infrablue camera
[98]	ODROID XU4, NVIDIA Jetson Xavier	Nighttime pedestrian detection systems for cars	YOLOv2	FLIR A325sc thermal camera
[95]	ODROID XU4, NVIDIA Jetson TX2	Collision checking for small aerial vehicles navigation	Custom pyramid-based spatial partitioning	FLIR thermal imaging camera
[75]	ODROID XU4	Computationally inexpensive misclassification minimization for aerial vehicles	Siamese Neural Network	D435i Depth Camera
[20]	NVIDIA Jetson Nano, NVIDIA Jetson AGX Xavier	Benchmark analysis of 3D object detection	Complex YOLOv3, Complex YOLOv4	USB attached video camera (Benchmark paper)
[18]	NVIDIA Jetson Nano, NVIDIA Jetson TX2, Raspberry PI4	Performance analysis of different hardware for object detection CNNs	Custom Deep-CNN	N/A (Benchmark paper)
[19]	NVIDIA Jetson TX1	Analysis of DNN architecture in image recognition	AlexNet, GoogLeNet, SENet, MobileNet	N/A (Benchmark paper)
[15]	Asus Tinker Edge R, Raspberry Pi 4, Google Coral Dev Board, NVIDIA Jetson Nano	Presentation and comparison of the performance of the presented systems in terms of inference time and power consumption	MobileNetV2, MobileNetV2 Lite, MobileNetV2 Quant. Lite	N/A (Benchmark paper)
[112]	NVIDIA Jetson TX2	Visual aid system for the blind via real-time object detection	CNN YOLOv2	Webcam
[125]	NVIDIA Jetson Xavier NX	Proposal of a fast and accurate method of power line edge intelligent inspection	RepYOLO, YOLOv5	UAV camera
[13]	NVIDIA Jetson Nano, Raspberry Pi 3	Early cardiovascular disease prevention through ultrasound	DNN (custom models for different tasks)	Ultrasound
[126]	NVIDIA Jetson Nano	Passenger safety monitoring	DNN (YOLO, SSD)	360◦ view camera
[3]	NVIDIA Jetson TX1	Production safety oversight in coal mines	FL-YOLO	Video surveillance camera
[14]	NVIDIA Jetson TX2	Traffic flow detection and management	YOLOv3, DeepSORT	Canon EOS550D camera
[172]	NVIDIA Jetson TX2	Improve the effectiveness of image captioning	Captioning. BDR-GRU	N/A
[115]	Raspberry Pi 4, NVIDIA Jetson Xavier	COVID Identification through chest CT scans	Anam-Net	CT Scanner
[191]	NVIDIA Jetson TX2, NVIDIA Jetson Nano	Latency estimation on embedded systems	AlexNet, VGG16 ResNet-50, MobileNetV2	N/A
[7]	Nvidia Jetson AGX, Raspberry Pi 4	Hand gesture recognition	Custom Deep CNN model	Thermal camera
[89]	Nvidia Jetson Nano, Nvidia Jetson TX2, Nvidia Jetson Xavier NX, Nvidia Jetson Xavier AGX	Facial recognition inference comparison between edge and cloud devices	MTCNN detector, FaceNet	None
[146]	NVIDIA Jetson AGX Xavier	Person detection using top clothing	Mask-R-CNN, YOLACT++	N/A
[5]	NVIDIA Jetson TX1, NVIDIA JetsonTX2	Lightweight real-time traffic light detection for autonomous vehicles	Lightweight Convolution Neural Network	AVT camera (only used for data collection)
[192]	NVIDIA Jetson Nano	Real-time video analysis for edge computing	Custom architecture consisting of Front-CNN and Back-CNN	Video camera
[193]	NVIDIA Jetson TX2	Low-power and real-time deep learning-based multiple object visual tracking	CNN-based custom architecture	5MP CSI camera
[114]	NVIDIA Jetson TX2	Localize veins from color skin images.	CNN	2-CCD multi-spectral prism camera (JAI AD-080-CL)
[78]	Raspberry Pi 3 B+, with or without a neural compute stick (Intel Movidius), NVIDIA Jetson Nano	Protect crops from ungulate attacks	YOLO, Tiny-YOLO	Camera module (Raspberry Pi)
[128]	NVIDIA Jetson TX2	Detecting and tracking sinkholes via video streaming	Cascaded CNN	Video camera
[2]	NVIDIA Jetson Nano	Analyze face structure from video feed and detect drowsiness from facial features	OpenCV facial recognition	Webcam camera
[154]	NVIDIA Jetson TX1	Detecting, tracking, and geolocating based on a monocular camera of an aerial drone	YOLOv3	Monocular Camera
[155]	NVIDIA Jetson TX2	Drone detection	YOLOv3	Spherical Camera (Ricoh Theta S)
[173]	NVIDIA Jetson TX2	Filter Pruning DNNs	VGG-16, ResNet-56, LeNet, FCNet-120	N/A
[156]	NVIDIA Jetson TX2	Resource constrained object tracking	CNN	N/A
[194]	NVIDIA Jetson AGX Xavier	Energy-efficient acceleration of deep neural networks	DNN	N/A
[1]	NVIDIA Jetson TX2	Road marking detection for autonomous vehicles	CNN	Camera
[195]	NVIDIA Jetson TX1	Semantic Segmentation for autonomous vehicles	DNN	N/A
[196]	NVIDIA Jetson TX2	Improve semantic segmentation performance in contexts of various sizes and types in diverse environments	Segmentation CNN	N/A
[90]	NVIDIA Jetson Nano	Face mask detection system	CNN	TGCAM-2000STAR camera
[96]	NVIDIA Jetson Xavier NX	Depth estimation	FastMDE custom model	monocular camera
[197]	NVIDIA Jetson TX2, Edge tensor processing unit, neural compute stick, and neural compute stick2	Fusion Pruning DNNs	DNN	N/A
[157]	NVIDIA Jetson TX2	Object detection and object tracking on drones with limited power and computational resources	CNN	Logitech BRIO camera
[79]	NVIDIA Jetson Nano	Detection of ripe coffee beans	CNN	Intel realsense depth camera D435
[84]	NVIDIA Jetson TX2	Intelligent pest detection	Tiny-YOLOv3	High-resolution optical drone camera
[97]	NVIDIA Jetson TX2	Personal fall detection system	Gaussian mixture model (GMM)	Image depth camera, RGB camera
[198]	NVIDIA Jetson TX2	Reduce computational complexity and memory consumption of CNNs architecture on low-power devices	Light-YOLOv4	N/A
[199]	NVIDIA Jetson TX2	Reduce computational complexity and memory consumption of CNNs architecture on low-power devices	CNN	N/A
[145]	NVIDIA Jetson Nano	Identify and detect suitable grasping point on objects for robotic limbs	ASP U-Net (DCNN)	A Basler acA2500-14uc industrial RGB camera with Computer M3514-MP lens
[100]	NVIDIA Jetson TX2	Lightweight road object detection for autonomous vehicles	CNN	Camera
[101]	NVIDIA Jetson Xavier	Lightweight Multitask object detection and semantic segmentation for autonomous vehicles	DCNN	N/A
[102]	NVIDIA Jetson Xavier NX	Path Planning for self-driving vehicles and robotic systems	LSTM	Camera
[103]	NVIDIA Jetson Nano	Thermal object detection for assisted driving	Thermal-YOLO	LWIR prototype thermal camera
[200]	NVIDIA Jetson AGX Xavier	Improve embedded system performance in autonomous vehicles	DNN	N/A
[171]	NVIDIA Jetson Xavier NX	Trajectory tracking for small drones	MPC	Velodyne Lite 16 Lidar sensor
[158]	NVIDIA Jetson TX2	Navigation for indoor autonomous drones	SSD	Fisheye lens on the PointGrey Firefly camera
[159]	NVIDIA Jetson TX2, NVIDIA Jetson Nano	Object detection via template tracking	OpenCV	N/A
[176]	NVIDIA Jetson Nano	Epileptic seizure detection	DNN	Electrocardiogram
[116]	NVIDIA Jetson Nano	Posture recognition system for medical surveillance	MobilenetV2, LSTM	RGB camera
[129]	NVIDIA Jetson TX2	Concrete damage detection on the surface of buildings	YOLO-v3	Logitech Camera
[80]	NVIDIA Jetson TX2	Crop recognition for robotic weeding	ResNet-10	Canon PowerShot SX150 IS camera
[130]	NVIDIA Jetson AGX Xavier	Railway defect detection	TensorRT	Camera
[147]	NVIDIA Jetson Nano	Real-time metro passenger volume enumeration	CircleDet	HD video recording camera
[160]	NVIDIA Jetson TX2	Target tracking amongst static and dynamic obstacles	Model Predictive Control (MPC)	Drone camera
[161]	NVIDIA Jetson TX2	Underwater object gripping point detection	real-time lightweight object detector (RLOD)	ZED binocular camera
[104]	NVIDIA Jetson Xavier NX	Road obstacle detection for vehicles	Siamese Neural network	20 Hz stereo camera
[162]	NVIDIA Jetson TX2	Intelligent weapons targeting system	YOLOv5	N/A
[203]	NVIDIA Jetson TX1, NVIDIA Jetson TX2, NVIDIA Jetson TK1	Review of assisted driving in resource constrained hardware	ADAS	N/A
[117]	NVIDIA Jetson TX2	Diabetes diagnosis	R-CNN with InceptionV2	Jetson TX2 onboard camera
[17]	NVIDIA Jetson TX2, NVIDIA Jetson Xavier NX, and NVIDIA Jetson AGX Xavier	Benchmarking NVIDIA Jetson systems for visual odometry of flying drones	VINS-Mono, VINS-Fusion, Kimera, ALVIO, Stereo-MSCKF, ORB-SLAM2 stereo, and ROVIO	N/A
[177]	NVIDIA Jetson TX2	Low-power multimodal data classification	DCNN	Stand-alone Dual-mode Tongue Drive System
[201]	NVIDIA Jetson TX1	Provide a less resource costly object detection model for embedded systems	Tiny-YOLO-V3, Tinier-YOLO	N/A
[143]	NVIDIA Jetson Nano	Power system cyber security	recurrent neural networks (RNN)	N/A
[99]	NVIDIA Jetson TX1	Traffic sign identification for smart vehicles	deep convolutional neural network (DCNN)	USB webcam
[202]	NVIDIA Jetson Nano	Efficient video understanding	Temporal Shift Module (TSM)	Video camera
[113]	NVIDIA Jetson TX2	Rescue of natural disaster survivors through drone object detection	YOLOV3, YOLOV3-MobileNetV1, YOLOV3-MobileNetV3	Zenmuse XT2 gimbal camera
[105]	NVIDIA Jetson AGX Xavier	Object detection and recognition and energy management for autonomous vehicles	Deep reinforcement learning (DRL), YOLO	N/A (can theoretically use onboard camera or radar)
[163]	NVIDIA Jetson AGX Xavier	Object recognition for unmanned surface vehicles	YOLOv4, Siamese-RPN	High-definition photoelectric vision sensor
[81]	NVIDIA Jetson TX2	Accurate weed detection for micro aerial vehicles	SegNet	Multispectral camera
[148]	Raspberry Pi 4	Smart Urban waste management	SSD MobileNetV2	Pi Camera
[149]	Raspberry Pi 4B	Garbage identification for recycling	MobileNetV3	Camera
[131]	Raspberry Pi 4 Model B	Biometric scan for entry control	Vein and Periocular Pattern-based Convolutional Neural Network (VP-CNN).	Raspberry Pi NoIR camera
[132]	Raspberry Pi 4	Real time fire detection	CNN	Camera
[174]	Raspberry Pi 3	Patient anesthesia monitoring	DNN	Electroencephalogram
[175]	Raspberry Pi 3	Human posture detection	Multi-Mapping Spherical Normalization (MMSN)	Wireless body sensors (motion sensors, inertial sensors)
[118]	Raspberry Pi 3 Model B+	Reading assistance for blind people	OCR CNN	Raspberry Pi camera module V2
[178]	Raspberry Pi 3	Driver behavior monitoring	DCNN	IMU sensor, Shimmer Version 3 wearable body sensors
[106]	Raspberry Pi 3 Model B+	Scalable and computationally cheap networks for autonomous driving	DNN	Raspberry Pi camera
[110]	Raspberry Pi 3 Model B+	Early skin cancer detection	CNN	IR camera
[179]	Raspberry Pi 3 Model B+	Smart Urban waste management	Keras	Ultrasonic sensor
[180]	Raspberry Pi 3B	Fault detection in AC electrical systems	ArcNet (CNN)	Photoelectric sensor
[22]	Raspberry Pi 4B	Space exploration landing site selection	SegNet, FCN	N/A (dataset acquired from images taken by the Mars HiRISE camera)
[181]	Raspberry Pi 3 Model B+	Target classification at road gates with radar	SVM	Radar
[123]	Raspberry Pi 3 Model B+	Activity recognition for medical monitoring and rehab	CNN	Wearable Sensor
[124]	Raspberry Pi	Sign language recognition	CNN	Thermal camera
[11]	Raspberry Pi 3+	Speed bump detection for autonomous vehicles	CNN	Raspberry Pi camera
[182]	Raspberry Pi 3B+	Human activity recognition	CNN	Wearable multimodal sensors
[164]	Raspberry Pi 3B+	Drone landing automation	DNN	Raspberry Pi v1.3 camera with a fisheye lens
[119]	Raspberry Pi	Cervical cancer prevention	PiHRME	PiCamera
[183]	Raspberry Pi 3B+	Speech recognition	EdgeRNN	Audio sensor
[140]	Raspberry Pi	CPU heat tracking	Adaptive learning	Infrared thermal sensor
[91]	Raspberry Pi 4	High accuracy facial recognition	EfficientNet-Lite (CNN-KNN)	Webcam
[4]	Raspberry Pi 3B, NVIDIA Jetson TX1, NVIDIA Jetson TX2	Psychological stress monitoring	KNN, SVM	Heart rate and accelerometer sensors
[10]	Raspberry Pi 3 model B	Image recognition for sea life	CNN-based animal recognition	Pi Camera v2.1
[87]	Raspberry Pi 3 model B	Facial biometric scan	LGHP	Pi camera
[133]	Raspberry Pi 3 Model B+, Intel Neural Compute Stick 2	Security surveillance	Mask R-CNN	Surveillance camera
[204]	Raspberry Pi 3B+, NVIDIA Jetson TX2	Scalable and computationally cheap networks for embedded systems	DNN, MobileNetv2	N/A
[82]	Raspberry Pi 4	Weed identification for herbicide	Varied, includes CNN and KNN	The Raspberry Pi camera module version 2.0 with an 8-megapixel Sony IMX219 sensor
[184]	Raspberry Pi 3 Model B	Motor fault diagnosis	CNN	Hall effect sensor
[185]	Raspberry Pi 4 Model B	Machine state monitoring	CNN	Vibration Sensor, Accelerometers
[12]	Raspberry Pi 4	Violent assault recognition	mobile CNN	Surveillance camera (no actual live testing)
[186]	Raspberry Pi	Asthma risk prediction	CNN, DNN	SDS011 air quality sensor
[165]	Raspberry Pi 3 Model B+	Image classification	MobiHisNet (based on MobileNet)	N/A
[92]	Raspberry Pi 4	Facial recognition and facial expression recognition	CNN	Logitech c270 camera
[166]	Raspberry Pi	Counting individuals within a given video feed	Hidden Makarov Model	Camera
[120]	Raspberry Pi 4 Model B	Dog health monitoring through posture analysis	Mask R-CNN	Smart camera network
[144]	Raspberry Pi 3 Model B	Pedestrian profile recognition	2-layer CNN	FLIR Lepton thermal camera
[94]	NVIDIA Jetson TX2	Lightweight facial recognition for embedded systems	Facial action unit	Camera
[8]	Raspberry Pi 3 Model B	Speech source identification	CNN	SSL sensors, microphones
[167]	Raspberry Pi	Fish recognition for underwater drones	LeNet, AlexNet, GoogLeNet	360 degrees panoramic camera
[153]	NVIDIA Jetson Nano	AI traffic light control	SSD algorithm	Raspberry Pi camera
[187]	NVIDIA Jetson Nano	Battery charge management	Long Short-Term Memory (LSTM)	GY169 current converter sensor module
[142]	NVIDIA Jetson Nano	Automobile fog lamp intelligent control	CN-FWR5	IMX219 camera
[21]	NVIDIA Jetson Nano, NVIDIA Jetson TX1, NVIDIA Jetson AGX Xavier	Benchmarking paper	PointNet	N/A
[168]	NVIDIA Jetson Nano	Identifying different plant species	AlexNet, ResNet50, and MobileNetv2, within Python’s Tensorflow framework	Photo camera
[150]	NVIDIA Jetson Nano	Car counter Traffic management	TeleBot API	Logitech c922 webcam
[111]	NVIDIA Jetson Nano	Diabetic ulcer detection	VGGNet, MatConvNet, and DenseNet	Thermal Camera
[151]	NVIDIA Jetson Nano	Smart city traffic management	MobileNetSSD and YOLOv4	Camera
[188]	NVIDIA Jetson TX2	Food quality analysis	Support Vector Machines (SVM), Naive Bayes, k-Nearest Neighbours algorithm (K-NN), Decision Tree, Random Forest, Logistic Regression, Neural Networks	Nuclear magnetic resonance spectrometer, infrared spectrometer
[121]	NVIDIA Jetson Xavier NX	Colonoscopy	Mobilenet	Colonoscopy camera
[83]	NVIDIA Jetson TX2	Loose fruit detection for oil palm	Faster R-CNN	Camera
[127]	NVIDIA Jetson TX2, NVIDIA Jetson Nano	Hard hat detection on construction site	Histogram of Oriented Gradients	Surveillance camera
[137]	NVIDIA Jetson TX2	Monitoring vehicle driver tiredness in real time	MobileNetV3	Infrared Camera
[152]	NVIDIA Jetson Nano	Visual garbage detection	MobileNetV3Lite	N/A (most likely a video Camera)
[189]	NVIDIA Jetson Nano	Pot plant species identification and watering needs monitoring	MOBILENET SSD V2	Capacitive Soil Moisture sensor, Water Level Sensor
[107]	NVIDIA Jetson Nano	Algorithm review for self-driving car navigation	SVM, ANN-MLP, CNN-LSTM	Mini camera IMX-219
[138]	NVIDIA Jetson TX2	Real-time security surveillance for acts of violence	Local Maximal Occurrence (LOMO), Crossview Quadratic Discriminant Analysis (XQDA)	RaspiCam camera, panoramic spherical camera
[139]	NVIDIA Jetson Nano, Raspberry Pi 3 Model B+	Rescue operation robot computer vision	Haar Cascade, YOLO Tiny	No IR filter camera, LiDAR, Raspi Cam NOIR V2.1
[134]	NVIDIA Jetson Nano	Security surveillance for abnormal activity detection	YOLOv5	Logitech C270 Camera
[93]	NVIDIA Jetson Nano, NVIDIA Jetson TX2	Facial ID for security	LFFD, ResNet50, SeNet50, LFFD+ ResNet50, LFFD+ SeNet50	Camera
[190]	NVIDIA Jetson Nano	Radio frequency ID recognition	Baseline LSTM, baseline CNN, baseline CNMN, CNN with ResNet, CNMN with ResNet	Universal software radio peripheral
[141]	NVIDIA Jetson Xavier NX	Real-time image processing for fusion diagnostics	Max-Tree Representation	Thermal image camera
[135]	NVIDIA Jetson Nano	Security surveillance for unusual behavior	2D CNN	HD camera
[136]	NVIDIA Jetson Xavier NX	Fire and smoke detection	YOLOv3	Camera
[122]	NVIDIA Jetson Nano	Travel assistance for the visually impaired	MobileNet, SSD	Optical RGB camera
[9]	NVIDIA Jetson TX1	Real-time pedestrian detection for autonomous vehicles	Modified YOLO v2 (Model H)	Zed Stereo camera
[169]	Nvidia Jetson Nano, Nvidia Jetson TX1, Raspberry Pi 4	Artistic photography aesthetic score prediction	YOLO-CNN, Mobilenet, multi-threaded aesthetic predictor	N/A
[108]	NVIDIA Jetson TX2	Real-time vehicle detection on embedded systems	EfficientDet-Lite, Yolov3-tiny	N/A
[109]	NVIDIA Jetson AGX Xavier	Uncertainty-based real-time object detection for autonomous vehicles	tiny YOLOv3, Gaussian YOLOv3	Camera
[170]	NVIDIA Jetson Nano	Underwater object detection	YOLO v3, YOLO Nano Underwater	N/A (visual camera in case of field testing)

## Abbreviations

The following abbreviations are used in this manuscript:

ADAS	Advanced Driver-Assistance System
AI	Artificial Intelligence
ANN	artificial neural network
API	Application Programming Interface
BDR	Break Down Rate
CNN	Convolutional Neural Network
CPU	Central Processing Unit
CSI	Camera Serial Interface
CT	Computerized Tomography
DCE	Data Circuit-terminating Equipment
DCNN	Deep Convolutional Neural Network
DNN	Deep Nerual Network
DRL	Deep Reinforcement Learning
DTE	Data Terminal Equipment
FCN	Fully Convolutional Network
FLIR	Forward Looking InfraRed
GPU	Graphical Processing Unit
GRU	Gated Recurrent Unit
IR	Infra-Red
KNN	K-Nearest Neighbors
L4T	Linux for Tegra
LFFD	Light and Fast Face Detector
LGHP	Local Gradient Hexa Pattern
LSTM	Long Short-Term Memory
LiDAR	Light Detection And Ranging
MDE	Monocular Depth Estimation
ML	Machine Learning
MLP	Multilayer Perceptron
MMSN	Multi-Mapping Spherical Normalization
MPC	Model Predictive Control
MTCNN	Multi-Task Cascaded Convolutional Neural Network
MoCap	Motion Capture
OCR	Optical Character Recognition
OS	Operating System
RAM	Random Access Memory
RAM	Random Access Memory
RCNN	Region-Based Convolutional Neural Network
RGB	Red Green Blue
RNN	Recurrent Neural Network
RPN	Region Proposal Network
RaDAR	Radio Detecting And Ranging
SDK	Software Development Kit
SSD	Single Shot Detector
SVM	Support Vector Machine
TPU	Tensor Processing Unit
TSM	Temporal Shift Module
UAV	Unmanned Aerial Vehicle
USB	Universal Serial Bus
VP-CNN	Vein and Periocular Pattern-based Convolutional Neural Network
YOLO	You Only Look Once
